# Discovery, nuclear properties, synthesis and applications of technetium-101

**DOI:** 10.1038/s42004-022-00746-9

**Published:** 2022-10-20

**Authors:** Erik V. Johnstone, Natalia Mayordomo, Edward J. Mausolf

**Affiliations:** 1 Innovative Fuel Solutions (IFS) LLC., North Las Vegas, USA; 2 Helmholtz-Zentrum Dresden-Rossendorf (HZDR), Institute of Resource Ecology, Dresden, Germany

**Keywords:** Nuclear chemistry, Nuclear fusion and fission, Inorganic chemistry, Nuclear fuel

## Abstract

Technetium-101 (^101^Tc) has been poorly studied in comparison with other Tc isotopes, although it was first identified over ~80 years ago shortly after the discovery of the element Tc itself. Its workable half-life and array of production modes, i.e., light/heavy particle reactions, fission, fusion-evaporation, etc., allow it to be produced and isolated using an equally diverse selection of chemical separation pathways. The inherent nuclear properties of ^101^Tc make it important for research and applications related to radioanalytical tracer studies, as a fission signature, fusion materials, fission reactor fuels, and potentially as a radioisotope for nuclear medicine. In this review, an aggregation of the known literature concerning the chemical, nuclear, and physical properties of ^101^Tc and some its applications are presented. This work aims at providing an up-to-date and first-of-its-kind overview of ^101^Tc that could be of importance for further development of the fundamental and applied nuclear and radiochemistry of ^101^Tc.

## Abstract (web summary)

^101^Tc is a lesser studied technetium isotope, but its unique nuclear properties and varying routes of production make it interesting for an array of applications where its shorter half-life can be exploited. Here, the authors review the discovery, nuclear properties, synthesis and separation, as well as applications of ^101^Tc, and point towards future research directions.

## Introduction

The element technetium, with atomic number (Z) Z = 43, was discovered in 1937 through the transnational collaboration between American particle physicist E. O. Lawrence and Italian radiochemists E. Segrè and C. Perrier^1^. It was serendipitously synthesised by Lawrence during the prolonged bombardment of a molybdenum (Mo) plate with deuteron particles. Segrè and Perrier, responsible for the subsequent radiochemical workup of the activated Mo plate, isolated a new material with chemical and nuclear properties unlike any of the known elements at the time. Determining that they had isolated a new element, Segrè and Perrier were credited with the discovery and later in 1947 coined the novel element technetium (Tc), derived from the Greek word for artificial, *τεχνητoζ*^2,3^. With the help of B. Cacciapuoti, the first isotopes of Tc to be identified from this initial experiment were ^95m^Tc (*t*_1/2_ = 61 d) and ^97m^Tc (*t*_1/2_ = 91 d)^4,5^.

Today, there are ~35 isotopes of Tc that are known with atomic masses (*A*) spanning from *A* = 85 to 120. None of the isotopes of Tc are stable, making it the lightest inherently radioactive element on the periodic table^6^. On Earth, ^99g^Tc (*t*_1/2_ = 2.11 × 10^5^ y) is the predominant naturally occurring Tc isotope where it is found in ultra-trace quantities due to the spontaneous fission of ^238^U (uranium), induced fission of ^235^U by neutron capture in U-bearing ores, and capture of cosmic-ray neutrons in Mo-ores^7,8^. However, the dominant sources of Tc are anthropogenic in origin and are artificially generated through nuclear transmutation reactions.

The most prevalent Tc isotope today is ^99g^Tc, which is primarily sourced from fission of both ^235^U (with a fission yield ~6.2%) and ^239^Pu in nuclear reactors and nuclear weapons detonations. Because of its high fission yield and long *t*_1/2_, ^99g^Tc is generated in appreciable quantities in the spent fuel where it accumulates^9^. Although current waste strategies focus on retention and controlled disposal of Tc^10,11^, legacy mitigation policies were less stringent and discharge of Tc-carrying effluent streams into the environment was a common practice^12^. Thus, the introduction of bulk Tc into the biosphere can be attributed to a combination of these various practices and actions^13^.

^99g^Tc is also formed via ^99m^Tc (*t*_1/2_ = 6.01 h), which serves as the backbone of the radiopharmaceutical industry, pertaining to diagnostic imaging, and presently constitutes for tens of millions of procedures worldwide annually^14^. From an applications perspective, ^99m^Tc is the most frequently used Tc isotope for commercial, industrial, and/or economic purposes. Despite Tc’s broad capacity in regards to coordination chemistry, catalysis, superconductivity, corrosion resistance, etc., the innate radioactivity associated with its longer-lived isotopes makes its handling and widespread use complicated^9^; thus, the use of its shorter-lived Tc isotopes (i.e., ^94m^Tc (*t*_1/2_ = 52.0 min), ^94g^Tc (*t*_1/2_ = 293 min), ^95m^Tc, ^95g^Tc (*t*_1/2_ = 20.0 h), ^96m^Tc (*t*_1/2_ = 51.5 min), ^96g^Tc (*t*_1/2_ = 4.28 d), ^99m^Tc, and ^101^Tc (*t*_1/2_ = 14.2 min) in radioanalytical or medical tracer studies and similar applications are the most commonly employed.

Of the aforementioned isotopes, ^101^Tc is comparatively one of the lesser discussed in the literature. However, its unique nuclear properties and varying routes of production make it an interesting Tc isotope nonetheless. Moreover, it is present in an array of applications, where its shorter *t*_1/2_ is exploited. The motivation behind this review was our recent work highlighting ^101^Tc production and isolation using a fusion-based compact accelerator neutron source^15^. Thus, a collection of some of the existing literature on ^101^Tc pertaining to its discovery, nuclear properties, synthesis, and application is provided in order to allow a better understanding of the isotope. With rapid developments being made in the areas of nuclear safeguards, medical isotopes, fusion energy materials, and novel fission technologies, it is plausible that ^101^Tc and the *A* = 101 isobar may see a mirrored growth in interest from the scientific community.

## Discovery of ^101^Tc

Nystrom and Thoennessen provide a comprehensive list of the known Tc isotopes and a brief description of their discoveries, including ^101^Tc^16^. They state that the discovery of ^101^Tc, was simultaneous amongst two groups in 1941: (1) Maurer and Ramm^17^, and (2) Hahn and Strassmann^18,19^. The credit for the discovery of ^101^Tc was given to Maurer and Ramm as well as concession by Hahn and Strassmann for identifying the puzzle of the near-identical half-lives of ^101^Mo and ^101^Tc. However, prior publications in the literature, which are actually alluded to in the work of Hahn and Strassmann^19^, point to evidence of the production and identification of ^101^Tc, contradicting this account.

Japanese researchers Sagane et al. reported in two publications from 1940 a new isotope of masurium (Ma), which was an unofficial name of Tc predating its current title, with a mass of *A* = 101^20,21^. Samples of Mo were activated with slow neutrons, and using an undisclosed, rapid separation the new Tc (Ma) isotope was isolated from its parent radionuclide ^101^Mo. Both half-lives were measured: for ^101^Mo, a *t*_1/2_ value of 19 ± 1 min was determined, whereas for ^101^Tc (^101^Ma) the value was 9 ± 1 min with a corresponding *β*^*−*^ energy of 1.14 MeV^20^. Likewise, Sagane et al. were able to determine that ^101^Tc (^101^Ma) was effectively produced by exposing Mo in a slow neutron field, but not a fast one whereas ^99^Ma (i.e, ^99m^Tc) was, which is consistent with what is known about these isotopes today (e.g., ^100^Mo(*n*, 2*n*)^99^Mo; ^99^Mo → ^99m^Tc)^22^. Therefore, although the works of both Maurer and Ramm and Hahn and Strassmann provided a more accurate measure of ^101^Tc according to its *t*_1/2_, Sagane et al. were the initial group to show isolation and preliminary data that assisted the field in its identification. It should be mentioned that the suggestion of a hypothetical ^101^Ma by Hahn and Strassmann in the late 1930s prior to its correct identification^23^ did aid in explaining the process of fission by Meitner and Frisch^24^.

Interestingly, Sagane et al. point out the recently discovered ^99^Ma (*t*_1/2_ = 6.6 h; i.e., ^99m^Tc) by Segré and Seaborg^25^ and make a comparative note about developing chemistry of the element using these newly discovered isotopes. They state the use of ^101^Tc (^101^Ma) would be more convenient for uncovering its chemistry due to its ease of production with slow neutrons for a short irradiation time (i.e., ~24 min) and its energetic *β*^−^ emission^20^. However, in retrospect, ^99m^Tc and ^99g^Tc became much more crucial in the development of Tc chemistry than ^101^Tc due to other technological advances as discussed earlier^9^.

## Nuclear properties

The isotope ^101^Tc is situated in the neutron-rich region of known Tc isotopes with 58 neutrons (*N*) for its 43 protons. Considering its mass, located in the *A* ~100 transitional region of nuclei shapes from spherical to well-deformed, the ground-state nuclei of the neutron-rich ^101^Tc is reported to have a higher degree of deformation in comparison to the lighter proton-rich ones, e.g., spherical, semi-magic ^93^Tc (*N* = 50)^26,27^, and lesser than the heavier ones, such as ^103^Tc (*N* = 60)^28^. For example, in the positive and negative parity bands of odd-*Z* Tc isotopes (*A* = 95–101), a systematic decrease was observed in level energies with the same spin according to increasing neutron number. Likewise, the level order inversion for the low-spin states in the *π*5/2^+^ state, following this same trend, reduces across ^95,97,99,101^Tc, finally disappearing for ^103^Tc. These phenomena were attributed to the increasing deformation parameter and decreasing Coriolis interaction consistent with the increase in neutron number^29^. Accompanying the increase in neutron number from *N* = 50 to 60, a transition from a near-spherical to a triaxial-deformed nuclei shape along with an increase in the quadrupole deformation was determined using Routhian surface (RTS) calculations. Triaxiality is predicted to initiate in ^97^Tc and gradually evolve throughout ^99,101,103^Tc^29^. It was determined that the shape transition of the ^101^Tc nucleus is similar to that of of ^101^Mo, where shape coexistence of the nucleus occurs. Due to the proximity of *Z* = 43 to the *Z* = 40 sub-shell closure, the strength of proton-neutron interactions can alter the effects of this sub-shell, thereby manifesting shape changes and coexistence^30^.

1 $${{\,\!}_{\,\;42}^{101}}{{{{{\rm{ M}}}}}}{{{{{{\rm{o}}}}}}}_{59}\mathop{\longrightarrow }\limits_{14.61\,\min }^{{{\beta }^{-}}}{{\,\!}_{\;\,43}^{101}}{{{{{\rm{T}}}}}}{{{{{{\rm{c}}}}}}}_{58}\mathop{\longrightarrow }\limits_{14.02\, \min }^{{{\beta }^{-}}}{{\,\!}_{\;\,44}^{101}}{{{{{\rm{R}}}}}}{{{{{{\rm{u}}}}}}}_{57}({{{{{\rm{stable}}}}}})$$

In the *A* = 101 isobar chain, ^101^Tc is directly preceded by its parent isotope ^101^Mo (*t*_1/2_ = 14.61 min) and is formed through *β*^−^ decay (Eq. 1)^31–33^. The decay occurs through the *J*^*π*^ = +1/2 ground-state transition of ^101^Mo to the *J*^*π*^ = +9/2 ground-state of ^101^Tc. Wiles conducted a study on low-level decays of ^101^Mo and ^101^Tc, and was unable to identify a p_1/2_ isomeric state for ^101^Tc^34^; however, an isomeric state of 760 ± 50 µs at *E*_*γ*_ = 192.0 ± 0.2 keV was induced via the (*γ*, *p*) photonuclear reaction with 26 MeV LINAC Bremsstrahlung on a ruthenium (Ru) target^35^, which was also observed by Bartsch et al.^36^. Meanwhile, high-spin structures of ^101^Tc have been studied up to *J*^*π*^ = +31/2 via its production as a fission fragment from the bombardment of ^176^Yb with ^28^Si at 145 MeV; backbending in ^101^Tc was shown to occur similar to that of ^102^Ru with the same rotational frequency and excitation energy^37^.

**Fig. 1:**
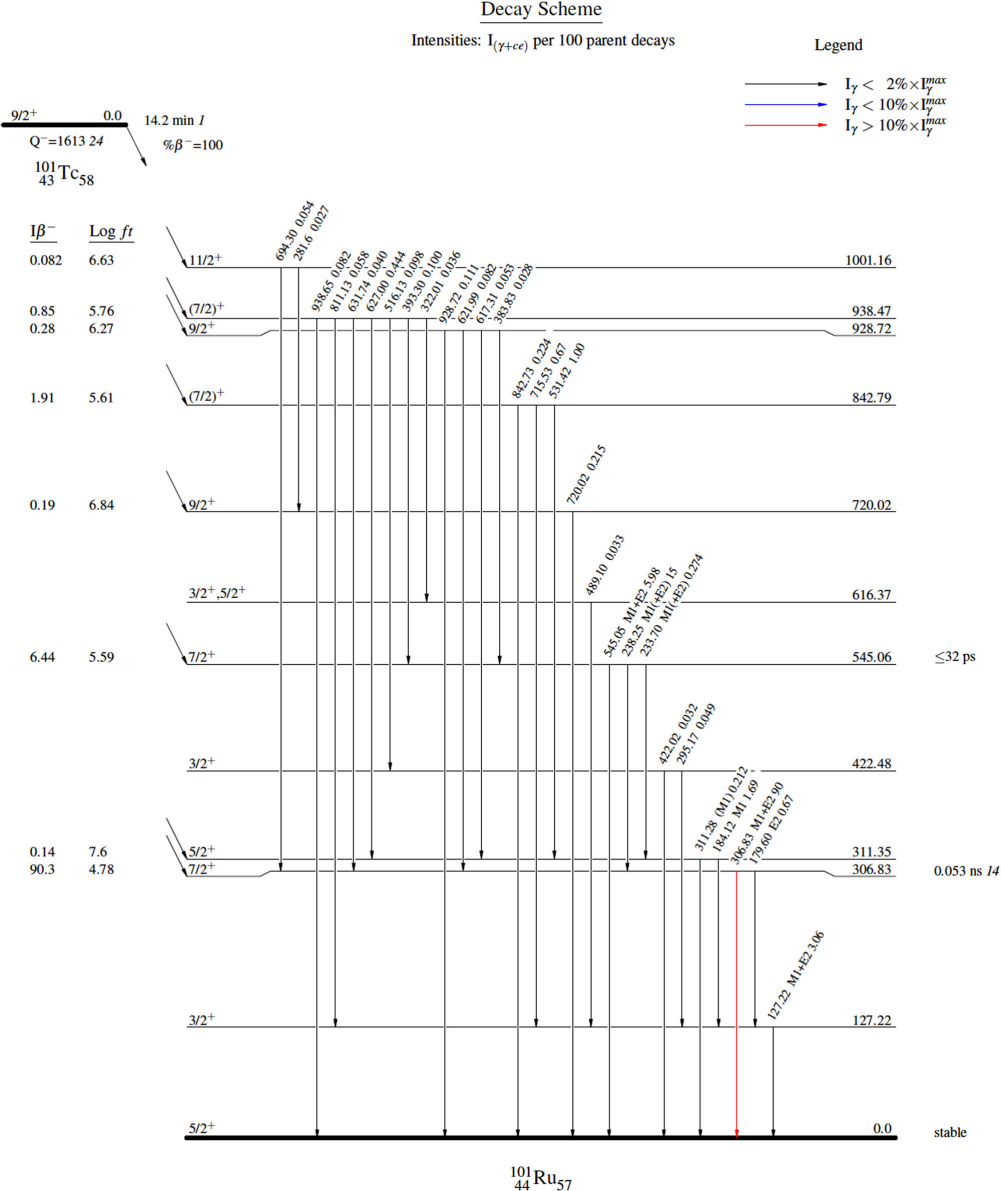
Decay scheme of ^101^Tc to ^101^Ru. Image generated via Evaluated Nuclear Structure Data File (ENDSF) database^33^.

As shown in Fig. 1, ^101^Tc decays by *β*^*−*^ emission, where it generates the stable end-member of the *A* = 101 isobar, ^101^Ru, with a transition from the *J*^*π*^ = +9/2 to the *J*^*π*^ = +5/2 state^33^. The *t*_1/2_ determination of ^101^Tc has been the topic of many studies as shown in Table 1^38–40^. The presently accepted value for the *t*_1/2_ of ^101^Tc is 14.22(1) min, which most closely resembles that determined by Abouzi et al., i.e., 14.224 ± 0.008 min^41^. However, more recent measurements have highlighted discrepancies in this value, and the measured *t*_1/2_ of ^101^Tc, which was free from ^101^Mo during the measurement, was 14.02 ± 0.01 min^42^. It is noted that in another recent experiment, da Silva et al. conducted measurements on 22 samples and resolved the *t*_1/2_ of ^101^Tc from the weighted results to be 13.725(13) min; the significantly smaller value compared to the tabulated one was attributed to the larger *t*_1/2_ measured for ^101^Mo, i.e., 14.893(13) min, being an interference^43^. The work of Hamida represents a much longer *t*_1/2_ in comparison to others, although no indication was provided for this discrepancy^44^. The most recent value of 16.0 min comes from Steinetz et al. in 2017, where ^101^Tc was generated via photon irradiation of deuterated lanthanide-Mo samples. Gamma-ray (γ) spectroscopy was performed using HPGe detectors, although no separation of parent-daughter isotopes was executed prior to measurements^45^. Considering all values collected here, the average *t*_1/2_ of ^101^Tc was determined to be 14.42 ± 1.92 min, or 1.39% difference from the reported value of 14.22 min.

**Table 1:** Experimentally measured values for the *t*_1/2_ of ^101^Tc for given production routes using different detectors.

Year Performed	Half-life, *t*_1/2_ (min)	Detector	Production route	Ref.
1940	9.0 ± 1	N/A	^100^Mo(n,γ)^101^Mo; ^101^Mo → ^101^Tc	Sagane^20^
1941	14.0	N/A	N/A	Maurer^17^
1942	14.0	N/A	U fission; ^101^Mo → ^101^Tc	Hahn^19^
1948	14.5	GM tube	^102^Ru(γ,p)^101^Tc	Perlman^38^
1948	16.5 ± 0.5	GM tube	^102^Ru(γ,p)^101^Tc	Mock^39^
1953	15.0	GM tube	^101^Ru(n,p)^101^Tc	Paul^68^
1954	14.3 ± 0.1	GM tube	^100^Mo(n,γ)^101^Mo; ^101^Mo → ^101^Tc	Wiles^34^
1957	14.0 ± 0.1	NaI	^101^Mo → ^101^Tc	O’Kelley^32^
1960	15 ± 3	Proportional counter	^103^Rh(n,^3^He)^101^Tc	Kumabe^40^
1990	14.22 ± 0.008	HPGe	^100^Mo(n,γ)^101^Mo; ^101^Mo → ^101^Tc	Abzouzi^41^
2008	14.02 ± 0.1	HPGe	^100^Mo(n,γ)^101^Mo; ^101^Mo → ^101^Tc	You^42^
2009	13.725(13)	HPGe	^100^Mo(n,γ)^101^Mo; ^101^Mo → ^101^Tc	da Silva^43^
2010	17.6 ± 3.5	HPGe	^100^Mo(n,γ)^101^Mo; ^101^Mo → ^101^Tc	Hamida^44^
2017	16.0	HPGe	^100^Mo(n,γ)^101^Mo; ^101^Mo → ^101^Tc	Steinetz^45^
*t*_1/2_ (avg)	14.42 ± 1.93			

The overall Q-value associated with the decay of ^101^Tc is roughly 1613 keV (Fig. 1). Table 2 shows the *β*^*−*^ emissions associated with ^101^Tc decay and their respective properties. The average *E*_β_ is ~473.6 keV and is made up of several distinct *β*^*−*^ emissions occurring at 225 keV (0.85%), 263 keV (1.92%), 385 keV (6.44 %), and 487 keV (90.3%). Its *β*_max_ of ~1320 keV corresponds to its highest energy *β*^*−*^ decay as shown in Table 2^33^. As previously stated, ^101^Tc decays into stable ^101^Ru, the end-member of the *A* = 101 isobar.

**Table 2:** *β*^*–*^ emissions associated with the ^101^Tc decay to ^101^Ru based on data from the ENDSF database^33^.

Avg. E_β-_ [keV]	Abs. I_β-_ [%]	Level energy [keV]	Max. E_β-_ [keV]	Log *ft*
210 *10*	0.82 *8*	1001.16 *15*	(612)	6.63 *8*
225 *10*	0.85 *2*	938.47 *5*	(675)	5.76 *6*
229 *10*	0.28 *1*	928.72 *5*	(684)	6.27 *6*
263 *10*	1.91 *5*	842.79 *5*	(770)	5.61 *6*
312 *10*	0.19 *1*	720.02 *6*	(893)	6.84 *7*
385 *11*	6.44 *16*	545.06 *4*	1070 *30*	5.59 *5*
485 *11*	0.14 *7*	311.35 *4*	(1302)	7.6 *3*
487 *11*	90.3 *2*	306.83 *4*	1320 *30*	4.78 *5*

Italicised numbers represent associated errors.

There are a host of characteristic *γ*-rays emitted during the decay of ^101^Tc to ^101^Ru as shown in Fig. 1 and Supplementary Table S1. Martin et al. was the first to study the *γ*-ray transitions associated with this occurrence, establishing 22 γ-rays^46^, and has since been the subject of multiple studies^41,47–53^. A more recent study by Hammed et al. catalogues 30 characteristic *γ*-ray peaks that were consistent with those reported in the literature, although not all of the ones reported previously were present in their investigation^31^. The dominant *γ*-rays associated with the decay of ^101^Tc and their respective absolute intensities per 100 decays are: 127.22 keV (2.63%), 184.12 keV (1.60%), 306.83 (89%), and 545.05 (5.96%).

## Production

Table 3 summarises the production routes for ^101^Tc addressed in this section.

**Table 3:** Examples of production routes for ^101^Tc.

Reaction	Source	Energy	Ref.
*Neutron-driven reactions*			
^100^Mo(n,γ)^101^Mo; ^101^Mo → ^101^Tc	Be-D accelerator	Thermal	Sagane^20,21^
	Fusion: D(d,n)^3^He	Epithermal	Mausolf^15^
	~2 MeV photon irrad. of ErD_2.8_ + C_36_D_74_ + Mo and HfD_2_ + C_36_D_74_ + Mo	Thermal, epithermal, fast	Steinetz^45^
	Fission reactor	Thermal	Hammed^31^, You^42^, Cook^47^, Wright^52^
	Am-Be tank	Thermal	Hamida^44^
	Hybrid accelerator (p,n)	Thermal, epithermal, fast	Daly^61^, Mayodormo^62^
^103^Rh(n,^3^He)^101^Tc	Fusion; T(d,n)^4^He	Fast; ~14.8 MeV	Gray^64^, Csikai^65^, Husain^66^,
^101^Ru(n,p)^101^Tc	Fusion; T(d,n)^4^He	Fast; 13.8-14.8 MeV	Gray^64^, Paul^68^, Lou^69^, Kasugai^70^, Kielan^71^
^102^Ru(n,d*)^101^Tc	Fusion; T(d,n)^4^He	Fast	Gray^64^, Kasugai^70^, Sakane^72^
^104^Ru(n,α)^101^Mo; ^101^Mo → ^101^Tc	Fusion; T(d,n)^4^He	Fast	Gray^64^, Lou^74^, Kasugai^75^
*Fission-based reactions*			
^235^U(n,f)^101^A; ^101^A → ^101^Tc	Neutron-induced	Thermalised	Hahn^19^, Kiso^77^, Elmaghraby^78^, Götz^102^
^239^Pu(n,f)^101^A; ^101^A → ^101^Tc	Neutron-induced	Thermalised	Srivastava^81^
^209^Bi(α,f)^101^A; ^101^A → ^101^Tc	α-induced	60 MeV	Rattan^83^
^248^Cm(SF,x)^101^A	Spontaneous fission	N/A	Götz^102^
*Light ion reactions*			
^100^Mo(p,γ)^101^Tc	Cyclotron; proton	up to 18 MeV	Daly^61^, Gagon^84^
^100^Mo(d,n)^101^Tc	Cyclotron; deuteron	up to 13 MeV	Randa^87^
^100^Mo(d,p)^101^Mo; ^101^Mo → ^101^Tc	Cyclotron; deuteron	12 MeV	Randa^88^, Wiles^76^, Habib^90^
^100^Mo(t,d)^101^Mo; ^101^Mo → ^101^Tc	Sputtering titanium tritide cones	14 MeV	Habib^90^
^98^Mo(ɑ,p)^101^Tc	Cyclotron; alpha	10–50 MeV	Levkovski^91^
*Fusion-evaporation reactions*			
^100^Mo(^7^Li,α2nγ)^101^Tc	Cyclotron	49 MeV	Dejbakhsh^30^
^93^Nb(^18^O,2α2p)^101^Tc	15UD pelletron accelerator	85–90 MeV	Agarwal^92^
*Photonuclear reactions*			
^102^Ru(γ,p)^101^Tc	Betatron; synchrotron	100 and 50 MeV	Perlman^38^

### Neutron-driven reactions

Neutron capture reactions provide the production of ^101^Tc through both direct and indirect processes depending on the target material being irradiated. To clarify, a direct route would be the formation of ^101^Tc directly from the interaction of the neutron field with the target material, whereas indirect would generate an intermediate radionuclide that in turn yields ^101^Tc from its decay. The most likely known pathway for producing ^101^Tc is indirectly via ^101^Mo. For example, neutron capture on ^100^Mo generates the parent isotope ^101^Mo, i.e., ^100^Mo(*n*, *γ*)^101^Mo, which subsequently decays to ^101^Tc as shown in Eq. 1. This indirect production route was first explored in 1940 by Sagane et al.^20,21^ as previously described.

Shown in Supplementary Fig. S1 is the ENDF/B-VIII calculated neutron capture cross-section for ^100^Mo up to *E*_*n*_ = 20 MeV. The thermal neutron capture-cross section of ^100^Mo is *σ*_*c*_ = 0.199 ± 0.003 b. For epithermal, intermediate, and resonance energy neutrons, there is an increased probability of interaction on ^100^Mo, and the resonance integral cross-section (*I*_c_) of ^100^Mo is approximately *I*_c_ = 3.76 ± 0.15 b^54–56^. The epithermal to thermal neutron capture cross section ratio is ~18.8.

For fast neutrons, the probability of neutron capture on ^100^Mo drastically decreases after *E*_n_ ~1 MeV, and the likeliness of other neutron-driven reactions, such as (*n*, 2*n*), (*n*, *p*), (*n*, *n*’), etc. begin to dominate^57,58^. In fact, Sagane et al. observed this behaviour by using lithium-deuterium (Li-D) fast neutrons to irradiate a ^100^Mo-containing source, which was enclosed in a cadmium (Cd) box filled with boric acid in order to remove the slow neutron component. They ascertained that the lack of measurable presence of a *t*_1/2_ similar to that identified for ^101^Ma (i.e., ^101^Tc), suggested that its formation was predominated by (*n*, *γ*) reactions on ^100^Mo^59^. Similarly, Mausolf et al. demonstrated the production of ^99m^Tc and ^101^Tc via (*n*, *γ*) reactions using a D-D neutron generator outputting 2 × 10^10^ n·s^−1^ 2.45 MeV fast neutrons that were scattered down into the epithermal and thermal regions in an aqueous ammonium heptamolybdate (AHM, (NH_4_)_6_Mo_7_O_24_·4H_2_O) target^15^. In general, when using the neutron capture route for ^99^Mo/^99m^Tc production, whether it be an accelerator, reactor, or another neutron source, the co-production of ^101^Mo/^101^Tc will occur when using targets that are of natural isotopic composition or contain fractions of ^100^Mo. Those responsible for commercial irraditations in the ^99^Mo/^99m^Tc industry generally view ^101^Mo/^101^Tc as non-problematic impurities, as they typically decay away within a relatively short period post-irradiation^60^.

Steintez et al. observed the production of ^99m^Tc and ^101^Tc from their respective Mo parent radionuclides via low-energy (~2 MeV) photon irradiation of deuterated lanthanide elements erbium (Er) and hafnium (Hf) mixed with Mo, which was used as a “witness material” for tracking initiated nuclear reactions, in a deuterated paraffin matrix, e.g., ErD_2.8_ + C_36_D_74_ + Mo and HfD_2_ + C_36_D_74_ + Mo. The irradiations produced neutrons with thermal to epithermal energies, neutrons in the 1.4–2.5 MeV fast range, and those with energies >10 MeV were also detected. Irradiations performed under similar conditions of the hydrogenated counterparts as control samples yielded no induced radioactivity in the Er, Hf, or Mo materials^45^.

Daly et al. presented the simultaneous production of ^101^Tc using secondary neutrons generated during the proton irradiation of an Mo target in a cyclotron in order to determine the contribution ^101^Tc formation either from (*n*, *γ*) or (*p*, *γ*) reactions on ^100^Mo in the target itself. For this, a secondary Mo target was placed outside of the incoming beam of the cyclotron and was irradiated with neutrons emitted from the primary target to determine the contribution arising from the (*n*, *γ*) pathway alone^61^. Similarly, Mayordomo et al. demonstrated the simultaneous production of ^101^Tc and ^99m^Tc during routine ^18^F[FDG] production on a low-energy biomedical cyclotron. Secondary, natural Mo targets were situated adjacent to the primary ^18^F target, where anisotropic neutrons are generated as a byproduct via the ^18^O(*p*, *n*)^18^F reaction specifically, and irradiated within the resulting neutron field^62^.

Production routes using alternative target elements other than Mo have been investigated, such as rhodium (Rh) and Ru. For Rh (Supplementary Table S2), the first report of the ^103^Rh(*n*, ^*3*^*He*)^101^Tc reaction was from Fervert in 1965^63^. Shortly after, Gray et al., using a Crockcoft-Walton accelerator with a D_2_^+^ beam to generate 14.7 MeV deuterium-tritium (D-T) neutrons with fluxes of 1 × 10^9^ to 5 × 10^10^ n·s^−1^, irradiated samples of Rh(OH)_3_ and RhCl_3_^64^. The *Q*-value of the reaction was determined to be −8.7 MeV and the measured cross-section was < 400 nb. The reported value by Csikai in the same year was similar for 14.7 MeV neutrons at *σ* = 1.3 ± 0.6 µb^65^. Husain et al. explored the ^103^Rh(*n*, ^*3*^*He*)^101^Tc reaction using 14.8 MeV neutrons with a yield of 10^10^–10^11^ n·s^−1^ generated in a 400 kV Cockcroft-Walton accelerator via the *T*(*d, n*)^4^He fusion reaction. The experimentally determined cross-section was 2.0 ± 0.6 µb at *E*_*n*_ = 14.8 MeV^66^. Using a Rh foil enriched in ^103^Rh and a Cockcroft-Walton D-T accelerator, Diksic et al. obtained the highest cross-section value at 14.6 MeV of 16 ± 7 nb^67^.

With fast neutrons ^101^Tc can be generated through both direct and indirect routes using Ru targets. For the direct method, the reaction ^101^Ru(*n*, *p*)^101^Tc was first investigated by Paul and Clarke in 1953, however, unintentionally. The nuclide they intended to generate was ^101^Ru via neutron bombardment on an Ru target, although it is now known the identified ~15 min *t*_1/2_ is attributed to ^101^Tc and ^101^Ru is stable^68^. Luo et al. later studied this reaction and the measured cross-sections were: 15.7 ± 2.0, 18.4 ± 2.7 and 22.0 ± 2.4 mb at 13.5 ± 0.2, 14.1 ± 0.2 and 14.8 ± 0.2 MeV incident neutron energies, respectively^69^. Taking into account other potential reaction pathways from Ru targets as presented in Table 3, the authors were able to more accurately assign contributions arising from each route to be 0.316σ(^102^Ru(*n*, d*)^101^Tc) + 0.17σ(^101^Ru(*n*, *p*)^101^Tc) reaction cross-sections. These values were consistent with those reported previously^70,71^ as shown in Supplementary Fig. S2, although it is noted that both the calculated cross-sections from ENDF/B-VIII and JEFF-3.3 tend to overestimate these values, especially JEFF-3.3 in the lower energy regime between ~6 and 15 MeV.

The reaction ^102^Ru(*n*, d*)^101^Tc is another direct route^72^ with a reported *Q*-value of −6.119 MeV. Using fast neutrons, the reaction cross-sections were determined to be ~0.63 ± 0.07 mb, 1.23 ± 0.10 mb, and 2.77 ± 0.1 mb at 13.5 ± 0.2 MeV, 14.1 ± 0.2 MeV, and 14.8 ± 0.2 MeV, respectively^69,73^. The experimental values measured for this reaction are underestimated by ENDF/B-VIII calculations and slightly overestimated for JENDL-5, although the JENDL-5 trends better in general (Supplementary Fig. S3).

For the indirect method, the ^104^Ru(*n*, *α*)^101^Mo reaction proceeds with a *Q*-value ~2.05 MeV^64^. The most recent experimentally derived cross-sections were determined to be 4.6 ± 0.5 mb at *E*_*n*_ ~13.5 MeV and 6.4 ± 0.2 mb at 14.8 MeV^74^. For the latter data point, it trends better with those from earlier reports, i.e., Paul et al. and Gray et al., than those reported by Kasugai et al. in a similar energy regime (Supplementary Fig. S4)^75^.

### Fission-based reactions

Concerning the library of knowledge on fission reactions and the formation of fission products, it is not the intention of this review to fully encompass all studies that have been performed. The majority of these are comprised of the fission of actinide elements that either occurs spontaneously or is induced by a bombarding particle, such as *n*, *p*, *d*, *α or γ*. Instead, the intent here is to showcase a handful of examples that demonstrate the production of ^101^Tc through fission-based means. Interestingly, the mass region in which *A* = 101 is encompassed, i.e., *A* ~ 96–106, exhibits enhanced fission yields of primary fission fragments, containing 82 neutrons or 50 protons, and their respective secondary fission fragments arising from reactions such as ^235^U(*n*, *f*), ^238^U(*γ*, *f*), ^235^U(*d*, *f*), ^238^U(*d*, *f*), ^233^U(*n*, *f*), and spontaneous fission of ^242^Cm (curium), due to fine structure associated with closed nuclear shells^76^.

The first report of the fission-derived *A* = 101 isobar ^101^Mo and/or ^101^Tc was Hahn and Strassmann, identifying it post-U fission^18,19^. Fission of U in a thermal or fast neutron flux forming these isotopes of interest has been the subject of several studies^77,78^. The neutron-induced fission yields from ^235^U of *A* = 101 are relatively abundant with ~5.17% and ~5.24% for thermal and fast neutrons, respectively; however, for 14 MeV neutrons the yield is slightly less (Table 4)^79^. For the *A* = 101 decay chain, ^101^Mo is preceded by ^101^Nb, but due to its shorter *t*_1/2_, i.e., (*t*_1/2_ = 7.1 s), it is typically not observed unless monitoring the reaction in real-time. It is also noted that the independent fission yield for ^101^Tc from ^235^U(*n*, *f*) is rather low, i.e., < 2.0 × 10^−4^%^80^, therefore in these types of experiments, contribution from this pathway is very little in comparison to the *A* = 101 isobar.

**Table 4:** Fission yield data for ^101^Tc originating from various fission sources via JEFF3.11^79^.

Isotope	*E*_neutron_	*σ*_fission_ (b)	Cumulative fission yield (%) of ^101^Tc
^235^U	Thermal (0.025 eV)	582.6	5.17
	Fast (1 MeV)	1.120	5.24
	14 MeV	2.053	3.78
^238^U	Fast (1 MeV)	0.014	6.43
	14 MeV	1.151	5.81
^239^Pu	Thermal (0.025 eV)	748.1	6.18
	Fast (1 MeV)	1.739	6.63

In another example, Srivastava et al. evaluated the yields of the heavier, neutron-rich isotopes of Tc including ^101^Tc from the thermal neutron-induced fission of ^239^Pu (plutonium); yields were compared with those obtained from the neutron-induced fission of ^235^U and the spontaneous fission of ^252^Cf (californium)^81^. The reported fission yields of ^239^Pu for thermal and fast neutrons for *A* = 101 are ~6.18% and 6.63%, respectively. In comparison to the photon-induced fission of ^240^Pu, the respective cumulative yield is relatively higher than those previously mentioned for ^239^Pu, where for photofission ^101^Mo and ^101^Tc account for the highest fission product yields amongst the lighter fission fragments^82^.

Rattan and colleagues demonstrated that ^209^Bi (bismuth) could be fissioned with high-energy *α*-particles generated in a variable energy cyclotron (VEC) to generate ^101^Mo^83^. The measured fractional cumulative yields of ^101^Mo were 0.655 ± 01.34% and 0.688 ± 0.114% at 55.7 and 58.5 MeV, respectively. The *σ*_fission_ values of the two different energies were comparable to each other, e.g., 3790 ± 242 µb at 55.7 MeV and 3640 ± 522 µb at 58.5 MeV.

### Light ion reactions (p, d, t, and α beams)

Several light ion reactions have been established for generating ^101^Tc using various particle accelerators. These include accelerators employing incident beams of *p*, *d*, *t*, and *α*. Typically, reactions of this nature are performed using high-energy cyclotrons, although alternative means of production have been identified. Under these circumstances, both routes, direct and indirect, for production of ^101^Tc are attainable depending on the target isotopics, beam type and beam energy employed.

Daly et al. irradiated ^100^Mo with 5-20 MeV protons and identified the ^100^Mo(*p*, *γ*)^101^Tc pathway^61^, which has been the subject of several studies. This pathway is of particular interest as it is a side reaction that occurs during the production of ^99m^Tc via (*p*, 2*n*) on ^100^Mo^84^. The calculated *Q*-value assigned to this reaction is +7.441 MeV, although it is reported that this reaction is rare for medium mass targets. The measurements performed by Daly et al. were consistent with nuclear calculations by Qaim et al. with a *σ*_max_ (~1 mb) at *E*_p_ ~14 MeV. However, more recent data from Lamere et al. and Gagon et al. show consistent, yet relatively lower *σ*_max_ than previously reported as shown in Supplementary Fig. S5^85,86^.

Alternatively, direct production of ^101^Tc can be accomplished via the (*d*, *n*) reaction utilising high-energy deuterons, i.e., ~13 MeV, on ^100^Mo with a corresponding *Q*-value of 5.2 MeV. Shown in Supplementary Fig. S6 is the experimentally measured cross-sections for this reaction between *E*_d_ = 3.9 and 11.3 MeV, where *σ*_max_ (~300 mb) occurs at *E*_d_ ~7.3 MeV. For *E*_d_ = 11.7 MeV, the thick target yield (*Y*) and production rate (*R*) were determined to be 10,378 µCi·µA^−1^ h^−1^ and 3493 µCi·µA^−1^, respectively^87^.

For the indirect production of ^101^Tc via ^101^Mo, Randa et al. measured the excitation functions for the ^100^Mo(*d*, *p*)^101^Mo reaction with deuteron beams < 13 MeV^88^. The resulting cross-section data for this reaction is shown in Supplementary Fig. S7, where the corresponding *Y* and *R* values were 6495 µCi·µA^−1^ h^−1^ and 2280 µCi·µA^−1^, respectively. In comparison to this experimental dataset, calculations via TENDL-2019 tend to underestimate reaction cross-sections with increasing energies ≥ 6 MeV. With the purpose of studying the nuclear structure of Mo isotopes such as ^101^Mo, Hjorth et al. have also reported the irradiation of Mo targets with 15 MeV deuterons^89^. Habib et al. performed irradiations of ^100^Mo with both a 12 MeV deuteron and a 14 MeV triton beam in order to target the ^100^Mo(*d*, *p*)^101^Mo and ^100^Mo(*t*, *d*)^101^Mo reactions, respectively, with the intention of studying the nuclear structure and spectroscopic states of ^101^Mo^90^.

Shown in Supplementary Fig. S8 is the experimental cross-section data for the ^98^Mo(*α*, *p*)^101^Tc reaction measured by Levkovski^91^. Under the conditions tested, the *σ*_max_ was measured at *E*_α_ ~24 MeV with a value of ~14.5 mb. In comparison to the TENDL-2019 calculations, the experimental cross-section data is notably higher for the corresponding energies than those provided by the calculation.

### Heavy ion fusion-evaporation reactions

Heavy ion fusion-evaporation reactions are another potential route for the production of either ^101^Tc or ^101^Mo that are usually performed at high-energies situated at or slightly above the Coulomb barrier for a particular heavy ion beam on a given target. These can be further characterised by complete and incomplete fusion reactions, depending on the degree/capacity for breakup fragments to fuse with the target. In general, incomplete fusion (ICF) or massive-transfer (MT) reactions proceed through a compound-nucleus-like de-excitement that yields an array of products by evaporation or γ-ray decay, similarly to that of the process of fission.

Dejbakhsh et al. studied high-spin states of ^101^Tc via the ^100^Mo(^7^Li, *α2nγ*)^101^Tc reaction^30^. Experimentally, this was completed by bombarding a metallic target of Mo with a 49-MeV ^7^Li beam in a high-energy cyclotron, and successively assessing the Z = 1 and 2 exit channels, i.e., ^101-104^Ru and ^101-103^Tc, with ^102^Ru and ^101^Tc yielding the highest statistics for the two channels, respectively. Agarwal et al. also reported the formation of ^101^Tc during the ICF dynamics of the high-energy bombardment of ^18^O on a ^93^Nb (niobium) target through the ^93^Nb(^18^O, *2α2p*)^101^Tc reaction pathway^92^. Once again, ^101^Tc is one of many residues that was formed under these circumstances. The excitation functions of this particular pathway are shown in Supplementary Fig. S9.

### Photonuclear reactions

Perlman et al. studied various photonuclear reactions on different elemental targets including Ru. For example, Ru metal was irradiated with photons produced using a betatron via the high-energy electron bombardment (i.e., 50 and 100 MeV) of a tungsten plate, and the formation of ^101^Tc was ascribed to the (*γ*, *p*) reaction on ^102^Ru^38^. As mentioned previously, both groups Uyttenhove et al. and Bartsch et al. employed high-energy linear electron accelerators (i.e., 32 and 65 MeV, respectively) to induce the ^102^Ru(*γ*, *p*)^101^Tc reaction^35,36^.

Although not a direct result of the photonuclear reaction itself, both ^101^Mo and ^101^Tc were reported during the photonuclear production of ^99^Mo in enriched ^100^Mo targets. It is likely that secondary neutrons liberated within the Mo target via the ^100^Mo(*γ*, *n*)^99^Mo reaction were captured within itself, in turn forming this radionuclidic pair^93^. For example, calculations corresponding to yields at the end of bombardment (EOB), the two most prominent radioisotopes aside from ^99^Mo were ^101^Mo and ^101^Tc using a 20.2 MeV, 45 µA beam over an 82-min irradiation^94^. A similar process was described by Tsechanski et al. when employing a one-stage approach for the photonuclear production of ^99^Mo using natural Mo targets. Under this scenario, ^99^Mo would be generated from two different nuclear transformation pathways: (1) the primary photonuclear reaction on ^100^Mo, and (2) successive neutron-capture on ^98^Mo from secondary neutrons generated as described above^95^.

## Separation

There exists a myriad of separation techniques for removing Tc from simple or complex mixtures of neighbouring host elements, such as Mo, Ru, Rh, etc.^9^. However, due to the short-half life of ^101^Tc, as well as its parent isotope ^101^Mo when not generated directly, it is necessary that the separation protocol implemented must be fairly rapid, i.e., approximately equivalent to one *t*_1/2_ of ^101^Tc^96^. Therefore, not all of the known options for separating longer-lived Tc isotopes from a host target material may be feasible for ^101^Tc. In this regard, there have been a number of viable separation platforms for isolating ^101^Tc that have been reported in the literature, including liquid-liquid extraction, chromatographic/solid-phase extraction, precipitation, and volatilisation (Table 5). Each of these methods will be expounded upon in the following section.

**Table 5:** Separation methods for obtaining ^101^Tc.

Target	Separation	Comments	Ref.
*Liquid–liquid extraction*			
MoO_3_	Org. phase: (C_6_H_5_)_4_AsCl in CHCl_3_; Aq. phase: 0.5 N NaOH and 14.8 N NH_4_OH	Continuous separation of [TcO_4_]^–^ into the org. phase from [MoO_4_]^2−^ in the aq. phase	Wright^52^
MoO_3_; dissolved post irradiation in HNO_3_ + H_2_SO_4_	Org. phase: 0.3 g·L^−1^ cetyldimethylbenzyl ammonium chloride in CHCl_3_; Aq. phase: 1 N NaOH	Tc extracted into org. phase as [TcO_4_]-complex	Aras^53^
Mo(CO)_6_	Org. phase: CHCl_3_; Aq. phase: 0.1 N HClO_4_	Recoil Mo/ Tc extracted into aq. phase with Mo–Tc-CO target in org. phase	Groening^100^
(NH_4_)_6_Mo_7_O_24_·H_2_O (50 g·L^−1^ soln.)	Org. phase: 40 g·L^−1^ α-benzoic oxime-ethyl acetate; Aq. phase: 1 M HNO_3_	Mo extracted into org. phase and Tc remains in aq.	You^42^
RhCl_3_ or RuCl_3_ in H_2_O; Rh(OH)_3_ in dil. HCl, neutralised with NaOH	Org. phase: CHCl_3_; Aq phase: 1% (C_6_H_5_)_4_AsCl/NaHCO_3_, pH = 8.5	[TcO_4_]^–^ extracted into org. phase with Tc carrier	Gray^64^
*Chromatographic/solid-phase extraction*			
N/A	Chromatography/sorption with HDEHP	Mo uptake on column; Tc remains in solution	Brodskaya^101^
(NH_4_)_6_Mo_7_O_24_·4H_2_O (~210 g·L^−1^ soln.)	Chromatography/sorption onto activated carbon (AC)	[TcO_4_]^–^ uptake on AC; Mo remains in solution	Mausolf^15^
*Precipitation*			
MoO_3_ (96.9% ^100^Mo)	Precipitation of Tc and Re carrier with (C_6_H_5_)_4_AsCl from NaOH/Na_2_S_2_O_8_ solution	~95% yields, within 15 min after EOB	Daly^61^
*Volatilisation*			
^235^U/^248^Cm via molecular planting	Reaction of flowing CO(g) with Tc and Mo fission products	Formation of ^101^Mo(CO)_6_; ~49% efficiency total	Götz^102^

### Liquid–liquid extraction

Liquid–liquid extraction is an efficient methodology for partitioning a mixture of elements where differences in charge and/or polarity of elements or complexes in solution can be exploited, for example, the use of polar aqueous and non-polar organic systems, which are immiscible or only moderately miscible with each other. When only small volumes of liquid are required to be processed and sufficient distribution coefficients (*K*_*d*_) for a system can be achieved, then liquid-liquid-based separations can be completed rapidly and on a continuous or near-continuous basis.

A predominant oxidation state of Tc is Tc(VII), where the hallmark compound under oxidising conditions is [TcO_4_]^−^, a chaotropic, low charge density complex that has the tendency to uptake into/onto non-polar solvents/mediums^97^. This behaviour makes it ideal for its recovery into non-polar solvents, which has been the key principle behind the low-specific activity ^99^Mo generator using methyl ethyl ketone (MEK) to remove [^99m^TcO_4_]^−^ from aqueous solutions of ^99^Mo-containing targets^98^.

Most literature using liquid–liquid extraction for isolating ^101^Tc reported employing CHCl_3_ as an organic solvent in conjunction with bulky coordinating cations, such as [(C_6_H_5_)_4_As]^+^ and [C_25_H_46_N]^+^, for removing the corresponding [TcO_4_]-salt from a caustic aqueous solution^50,51^. For example, while exploiting the varying chemistries between Ru/Rh and Tc, Gray et al. were able to perform quick separations on the order of ~12 min to isolate ^101^Tc from the corresponding target material. A ^99m^Tc/^99g^Tc tracer was used to track separation efficiencies, which varied between 50 and 80%, where ^101^Tc was extracted into an organic phase of CHCl_3_ from an aqueous solution of the dissolved Ru/Rh trichlorides targets containing 1% (C_6_H_5_)_4_AsCl. Subsequent analyses of ^101^Tc were performed on the isolated organic phase, in which Tc is found as the complexed form (C_6_H_5_)_4_As[TcO_4_]^64^.

Using comparable extraction conditions, e.g., (C_6_H_5_)_4_AsCl in CHCl_3_ and KBrO_3_/HNO_3_, Hild et al. employed a chemistry device called a MicroSISAK for isolating short-lived Tc isotopes, ^101^Tc and ^104^Tc^99^. The MicroSISAK is a micro-membrane extractor that was constructed for the purpose of continuously isolating short-lived, heavy elements such as bohrium (Bh). The Tc isotopes were generated online during the neutron-induced fission of ^235^U in a TRIGA reactor and transported with He/KCl gas jet from the target to the device. The device contains a micro-mixer element that intimately contacts the organic and aqueous phases prior to phase separation with a hydrophobic Teflon membrane. The extraction yield at 40 °C was 76 ± 1% for ^104^Tc, which was comparable for similar experiments performed with ^99m^Tc with 83 ± 3%^99^.

You et al. demonstrated the isolation of ^101^Tc from an irradiated aqueous AHM target using liquid–liquid extraction^42^. The irradiated solution was acidified with HNO_3_, after which the bulk Mo target material was removed using a solution of α-benzoic oxime-ethyl acetate as the organic phase. The Tc daughter remained in the acidified aqueous phase and was extracted again with the organic phase to remove any remaining Mo (i.e., ^93^Mo, ^99^Mo, and ^101^Mo) prior to performing subsequent measurements to ensure only ^101^Tc was present. The experimental design was aimed at maximising ^101^Tc yield and purity, so a quick irradiation in combination with a rapid separation protocol was adopted.

Experiments by Groening and Harbottle on “hot atom chemistry,” or the recovery of recoil products via radioactive decay or activation using a nuclear reactor or accelerator, investigated the separation ^101^Mo and ^99^Mo from a host matrix of Mo(CO)_6_ post-neutron bombardment^100^. Retention of ^101^Mo in the host phase was upwards of ~60% post-irradiation, however, effects of isochronal annealing (i.e., 10 min) of the target material prior to liquid-liquid extraction showed correlative increases in ^101^Mo retention upwards of ~85% at an annealing temperature of 120 °C. The separation of ^101^Tc/^99m^Tc from an irradiated Mo(CO)_6_ matrix containing ^101^Mo/^99^Mo was also performed. Tc isotopes were removed in the aqueous solution, where no stable partition between the organic and aqueous phases could be established. This phenomenon, which was more appreciably observed for ^101^Tc, was attributed to likely instability, such as hydrolysis, of a Tc-CO and/or mixed Tc–Mo-CO species in the presence of an aqueous HClO_4_ environment. For ^99m^Tc, the data showed that ~59% of it present post-irradiation was in a non-volatile form, which also indicates potential for separation of Tc from Mo(CO)_6_ via volatilisation^100^.

### Ion exchange (IE)/extraction chromatography

The use of IE or extraction chromatography can be a robust and rapid technique for isolating a single elemental fraction from a heterogeneous mixture of elements in solution. This technique is widely used for isolating short-lived radioisotopes in heavy element chemistry, radiopharmaceutical applications including positron emission tomography (PET), and for front or backend refinement in neutron activation analysis (NAA). In regards to ^101^Tc there are several accounts on the use of IE/extraction chromatography for its isolation.

For example, Brodskaya reported irradiating a sample of Mo with neutrons, forming both ^99m^Tc and ^101^Tc. A rapid extraction of these Tc isotopes from an aqueous Mo solution was completed using column chromatography. From the record, it is not certain what the stationary phase was composed of, although it is suspected that HDEHP on an inert support matrix was used^101^. Under these conditions, HDEHP is selective for Mo, and it is likely the resulting Tc is eluted from the column as in a standard radionuclide generator. Mausolf et al. reported the isolation of ^101^Tc and ^99m^Tc from low-specific activity ^101^Mo and ^99^Mo, respectively, using activated carbon (AC) to remove carrier-free Tc from an aqueous, acidic solution of AHM^15^. The separation was performed immediately after a ~15 min neutron irradiation by passing the AHM solution containing ^101^Mo/^101^Tc over a small column of AC, which was subsequently washed and isolated for successive characterisation. Several physicochemical mechanisms behind the uptake of Tc onto AC have been reported, such as electrostatic interactions, physisorption, chemisorption/ion-exchange, etc. For example, the protonation of AC surface functional groups at pHs below the point of zero charge (pzc), where carboxylic, carbonyl, laconic, and phenolic groups provide positively charged R–C=O and R–C–OH moieties, helps facilitate the AC sequestration of Tc as [TcO_4_]^–^.

### Precipitation

The use of radiochemical separations via precipitation is a fairly common, straightforward procedure where the insolubility of one element or compound is exploited over another. However, one drawback to this technique is that a carrier-agent is typically added to promote precipitation and to ensure full recovery of the radioisotope, which typically exists in very minute quantities according to mass. When a stable isotope of the chemical element of interest is available, then this is suggested for use, but if there is no stable isotope, such as for Tc, then a homologous element is considered, i.e., rhenium (Re), or one with similar chemistry may be an alternate option. If it can be handled in appreciable quantities, then a long-lived isotope such as ^99g^Tc is also technically feasible.

Daly et al. reported the use of precipitation as a means of separating ^101^Tc from a proton-irradiated sample of ^100^Mo via the (*p*, *γ*) reaction^61^. A short irradiation was performed, i.e., 15 min, on enriched MoO_3_ (96.9% ^100^Mo) and the resulting target material was dissolved in a mixture of NaOH and Na_2_S_2_O_8_. Following dissolution, the liberated ^101^Tc was recaptured via precipitation with a Re-carrier and (C_6_H_5_)_4_AsCl in the form of (C_6_H_5_)_4_As[ReO_4_]. Isolated samples with 95% yields were produced within one *t*_1/2_ of ^101^Tc.

### Volatilisation

The elements Tc and Mo, as well as some of their neighbouring elements Ru and Rh, are able to form various volatile compounds according to the chemical and physical environment. By exploiting the differences in vapour pressures of elements or compounds of interest, selective volatilisation can potentially be employed for separating them. In particular, oxide, halide, oxyhalide, and carbonyl complexes of Mo and Tc are well-known for their volatile nature^9^.

Götz et al. have demonstrated the atom-at-a-time production and separation of the *A* = 101 chain member ^101^Mo via the neutron-induced fission of ^235^U and spontaneous fission of ^248^Cm. The application of flowing N_2_/CO(g) at elevated temperatures and pressures over the fission-based targets allowed generation of isotopes of Mo as the volatile, mononuclear Mo(CO)_6_ complex and their continuous separation^102^. The total efficiency (ε_tot_) of ^101^Mo(CO)_6_ was determined to be 49%. Because Tc does not form a labile mononuclear CO complex, but rather a binuclear one Tc_2_CO_10_^103^, it is typically disregarded for atom-at-a-time production and separation^104^.

In general, the breadth of known separation methods for ^101^Tc is underwhelming in comparison to those that have been applied for other Tc isotopes, in particular ^99m^Tc and ^99g^Tc. Because of the commercial application of ^99m^Tc and the implications of ^99g^Tc removal from spent nuclear fuel, most of the separations knowledgebase of for Tc have focused on its isolation from low-specific activity Mo targets for ^99m^Tc and fissioned uranium (U)-based materials for ^99g^Tc^105^. For the latter, recovery of Tc from fission-generated feedstocks can be performed using a miscellany of techniques such as ion exchange resins (i.e., Purolite®-A520E/A530E/A532E, Amberlite^TM^-IRA-400, TEVA®, SuperLig®−639, ABEC, and Reillex^TM^ HPQ), cationic inorganic frameworks (layered double hydroxides (LDH), etc.), or metal organic frameworks (MOFs) to name a few, none of which have yet been applied for ^101^Tc^105–107^. However, the viabilities of these separation methods will be dictated on the ability to streamline processing of a fission-based target and reduce competition and co-extraction of other fission products. Thus far, selective and real-time volatilisation of Mo or Tc as recoiled fission products from the fission source has proven most effective for isolating minute quantities of ^101^Mo/^101^Tc^102^.

Of the separation pathways presented here, the majority are focused on the use of Mo targets as the source of transmuted ^101^Tc. A variety of techniques have been explored for isolating Tc from Mo targets under the circumstances for ^99m^Tc radiopharmaceuticals^108,109^. These include many of the same presented here for ^101^Tc, such as liquid–liquid extraction, column chromatography, volatilization, etc. Interestingly though, it was not apparent that many of those that have been thoroughly investigated for ^99m^Tc have also been tested for ^101^Tc. For example, liquid-liquid extraction using MEK as the organic extractant for [TcO_4_]^–^^110^; chromatography using high sorption capacity resins for Mo (meso- or nano-based inorganic supports such as Al_2_O_3_, TiO_2_, CeO_2_, etc.)^111^; chromatography based on Tc sorption resins (i.e., ABEC-2000, Analig® Tc02, Chemmatrix, Dowex-1×8, etc.)^112^; molybdate gel generators^113^; and thermal chromatographic volatilization^114^. Exceptions are the use of HDEHP, AC, and variations on the use of Mo(CO)_6_. Separation methods such as carrier-added precipitations, which are typically not used for ^99m^Tc production, are still a commonly employed radiochemical technique. However, the depth of work that has been reported for recovery of ^101^Tc via precipitation is not obviously extensive, and other bulky precipitating agents for [MO_4_]^–^ (M = Tc, Re) that could provide similar results include tetraphenylpyridinium (TPPy), tetraphenylphosphonium (TPP), tetrabutylammonium (TBA), etc., or exploiting the limited solubilities of reduced valent states of Tc/Re, such as for MO_2_ and MS_2_ (M = Tc, Re), and other precipitating or redox agents, i.e., Fe(OH)_2_^9,115^.

For alternative production routes via Ru or Rh targets, only liquid-liquid extraction was experimented for the isolation of ^101^Tc. Separations of fission-derived Tc, Ru, and Rh from each other are well-studied under the context of fuel reprocessing as well as is separating transmuted Ru from bulk Tc targets for waste processing; however, it appears that the study of separating trace Tc from bulk Ru or Rh is less known. The account from Gray et al. reported here demonstrates the ability to exploit the varying chemistries of the elements, i.e, [TcO_4_]^–^ versus M^3+^ (M = Ru, Rh), which could also be leveraged for other separation techniques^64^.

## Applications

### Neutron activation analysis (NAA) of Mo samples

NAA has been a technique heavily employed for the determination of trace quantities of Mo in samples of inorganic or organic origin. In this context, the use of the ^101^Mo/^101^Tc radionuclide pair can be an alternative to the ^99^Mo/^99m^Tc pair for NAA. In 1958, Fukai and Meinke introduced the use of this radionuclidic pair for NAA for determination of Mo content in marine samples via the separation and measurement of ^101^Tc. Separations were performed using (C_6_H_5_)_4_AsCl and detection of the 307 keV characteristic *γ*-ray was used^116,117^. For example, Diksic et al. also demonstrated that the shorter-lived ^101^Mo/^101^Tc chain could be utilised to measure the Mo content in bovine liver and orchard leaves^118^. NAA for the analysis of Mo in plant material was performed by van Zanten et al. via ^99^Mo and ^101^Mo. For ^101^Mo, samples (dried clover, ~0.5 g) were irradiated in a thermal neutron flux of 1.4 × 10^1^ n·cm^−2 ^s^−1^ for 20 min, dissolved in a sulfuric acid medium, and the ^101^Mo was recovered with a Mo carrier using liquid-liquid extraction in tri-*n*-octylamine in kerosene over a 15 min period. The entire process required ~1 h and the detection limit for Mo using ^101^Mo was 0.1 µg of Mo with a standard error of ~35%, which was qualified using values determined by ^99^Mo NAA and spectrophotometry^119^. Other biological materials that have been investigated for Mo content are human hair^120^, wool^120^, and urine^121^.

Sun et al. applying the ^101^Mo/^101^Tc pair in combination with pre-concentration via co-precipitation with thionalide–ammonium pyrrolidinedithiocarbamate from high pH solutions, were able to determine the concentration of Mo in the form of molybdate [MoO_4_]^2−^ in natural waters by NAA^122^. The detection limit of [MoO_4_]^2^^−^ was determined to be about 1 ng·L^−1^ with ppb to sub-ppb detection limits for natural water samples. The stated advantages were less interferences in the spectra or yield calculations in comparison to the ^99^Mo/^99m^Tc system (i.e., ^59^Fe, *E*_γ_ = 143 keV; ^235^U(*n*, *f*)^99^Mo;^102^Ru(*n*, *α*)^99^Mo), shorter irradiation/measurement times, higher sensitivities with lower background, and safer handling due to less activation in the samples.

Hetherington proposed and investigated the use of NAA to determine the enrichment of Mo targets for ^99^Mo zirconium molybdate gel generators^123^. The intent was to determine concentrations of ^98^Mo, ^100^Mo, and ^92^Mo via their activation products and corresponding radionuclidic daughters when applicable, i.e., ^99^Mo/^99m^Tc, ^101^Mo/^101^Tc, and ^93^Mo (*t*_1/2_ = 4000 y). Simulated samples were synthesised by mixing weighted quantities of enriched ^98^MoO_3_ (96.8% ^98^Mo) with natural isotopic MoO_3_ in various ratios, and the samples were irradiated for durations of minutes to hours with similar cooling periods depending on the radionuclidic pair to be analysed. This technique was shown to be effective with an accuracy of ±2% for determining the isotopic abundances of ^98^Mo and ^100^Mo in enriched samples, whereas the resulting activation was insufficient to collect appreciable data on ^93^Mo.

### Fission monitor in nuclear reactors and materials interrogation

Because of its association with fission-based processes, the ^101^Mo/^101^Tc radionuclidic pair and *A* = 101 isobar end-member ^101^Ru can serve as unique signatures and dialogistic tools^78,124,125^. For instance, Tohmay et al. were able to measure and differentiate fission product yield ratios of short-lived fission products to certify the enrichment of a natural U sample via thermal neutron fission of ^235^U and fast neutron fission of ^238^U; an Am-Be neutron source was used as a source of neutrons coupled with delayed *γ*-ray measurements on a HPGe detector for analysing fission products. Using this method, variations in fissile and fertile compositions (i.e., enrichment and depletion) could be measured, while specific fission product radionuclides including ^101^Tc, along with ^88^Kr, ^91^Sr, ^92^Sr, ^92^Y, and ^105^Ru, were determined to be prominent signatures of enrichment^124^. The authors suggested that a small, portable neutron source, such as the Am-Be source used in the study or one similar, could be implemented for nuclear materials interrogation by applying these techniques.

In the nuclear reactor environment, the use of *γ*-ray analysis of select fission products as a complementary method with delayed neutron measurements for monitoring potential fuel failure was performed in the 30 MW HANARO reactor in Korea. Several Tc isotopes originating from fission were detected in the coolant water: ^99m^Tc, ^101^Tc, and ^104^Tc with concentrations of 4.73 × 10^3^ Bq·L^−1^, 1.95 × 10^4^ Bq·L^−1^, and 8.35 × 10^3 ^Bq·L^−1^, respectively. Considering *t*_1/2_, decay scheme, peak area, and potential interferences, ^101^Tc was chosen for its 307 keV *γ*-peak, amongst other fission radionuclides, which could be measured with relatively small uncertainties despite the presence of a large Compton continuum located throughout the energy regime in the area of interest. The methodology proved to be beneficial as it was able to identify a very small fuel defect during normal reactor operation that would typically go undetected by the fuel failure detection (FFD) system based on delayed neutrons^126^.

A radioanalytical method that has been employed for determining the origin of atmospheric releases in fission-based accidents is measuring the isotopic ratio of non-naturally occurring Ru isotope pairs. For instance, sources of Ru generated from fission are typically depleted in the lighter Ru isotopes (i.e., ^96^Ru, ^98^Ru, ^99^Ru, and ^100^Ru), due to formation of stable Mo or long-lived Tc within these isobars, and enriched in heavier ones (i.e., ^101^Ru, ^102^Ru, and ^103^Ru), which are preceded by isotopes with relatively short half-lives. It was shown that ratios of ^100^Ru/^101^Ru and ^102^Ru/^101^Ru were higher for material originating from civilian power reactors due to contributions arising from fission of ^239^Pu in-grown in later fuel cycles, whereas these ratios were lower in Hanford groundwater where ^235^U fission was dominant over ^239^Pu^127^.

The use of the *A* = 101 isobar in combination with other isotopic markers has been key for developing an understanding of geological transformations and geochemical behaviours of different isotopes and radioisotopes that were formed in the Oklo natural fission reactor in Gabon. For example, one study on the micro-metallic and ε-phase aggregates, including the fission-derived elements Ru, Rh, Pd, Te, Pb, As, S, and Bi, were collected and the isotopic ratios of different isotopic systems, i.e., ^235^U/^238^U, ^90^Zr/^91^Zr, ^95^Mo/^97^Mo, and ^99^Ru/^101^Ru, were analysed^128^. Whereas the ^90^Zr/^91^Zr and ^95^Mo/^97^Mo ratios in the metallic aggregates varied little in composition, likely due to constant separate mixing of fissiogenic and non-fissiogenic components, the ^99^Ru/^101^Ru ratios exhibited large variations, which could not be described by such processes. On one hand, it was suggested that chemical fractionation between Tc and Ru during reactor criticality had occurred, where long-lived ^99g^Tc and quickly formed ^101^Ru would have behaved dissimilarly under dynamic redox conditions arising from the radiolysis of water. It is suggested that fractionation would have occurred over the timeframe of the *t*_1/2_ of ^99g^Tc or ~2.11 × 10^5 ^y, where the leaching behaviour of Tc, said to be 10^3^–10^4^ times that of Ru, would have been a strong driver of fractionation between the two elements. Likewise, the chemical fraction of Tc and Ru could have taken place during the incorporation of each into the metallic aggregate^128^. On the other hand, Groopman *et* al. attributed enrichments of the ^99^Ru/^101^Ru ratio to the propensity of the ^99g^Tc(*n*, *γ*)^100^Tc reaction to proceed, which would have occurred under the high neutron flux of the reactor ~1.2 × 10^21^ n·cm^−2^ s^−1 ^, post-Tc sequestration^129^. Under the scenario of Tc mobility within the uraninite or in other phases, a more even distribution of ^100^Ru throughout the particles would have been observed, which was not the case. Thus, it was suggested that if chemical fraction via leaching of Tc from the ε-phase had occurred, then it was likely not significant in extent and would have occurred when most Tc had already decayed to Ru.

### Uranium-molybdenum (U-Mo) fission reactor fuels

Low enriched uranium (LEU)-Mo alloy dispersion and monolith fuels, i.e., LEU-10%Mo, have been implemented as an alternative to high enriched uranium (HEU) fuels for fission reactors. The desirable refractory behaviour of Mo combined with its high solid solubility in the gamma (*γ*) bcc phase of U makes it a viable candidate for generating a robust, high-temperature, higher-density alloy^130,131^. The isotopics of the Mo used in the fuel, however, are non-negligible, where the average neutron capture cross-section is σ_c_ = 2.57 b for natural Mo, a value 13-times larger than that of zirconium (Zr), a common structural material used in the core of thermal reactors. Therefore, in order to increase efficiencies in fuel burn-up, geometry, composition, and overall neutronics, the use of enriched Mo fractions has been proposed. The isotope ^95^Mo constitutes the second most naturally abundant Mo isotope (i.e., 15.9%), and it has the highest neutron cross-sections for both thermal and epithermal neutrons, i.e., *σ*_c_ = 13.6 b and *I*_c_ = 117 b. The use of either light or heavy enriched fractions of Mo depleted in ^95^Mo has been proposed, where the average absorption cross-section is reduced by a factor of 2 when using the heavy fraction (i.e., ^96-100^Mo)^132^. Centrifugal enrichment of ^98^Mo and ^100^Mo for the commercial production of medical isotopes is an ongoing process; thus, this could be a potential source for providing enriched Mo for U-Mo fuel fabrication^133^.

In relation to ^101^Mo/^101^Tc, it is noted that whether using natural Mo or a heavy-enriched fraction in U-Mo fuels, the formation of this mother-daughter isotopic pair will occur due to neutron capture on the Mo component of the fuel. As previously discussed, the *A* = 101 isobar is also a product of fissioning actinides, and in the context of scenarios where fission-rich processes take place, this will become another source of ^101^Mo/^101^Tc. On one hand, this radionuclidic pair could become a potential interference if the *A* = 101 isobar is used a fission signature, whereas on the other hand, if the neutronics and the production rate of ^101^Mo/^101^Tc through neutron capture on Mo are well-known under the circumstances of interest, then the added Mo could serve as a secondary internal reference for comparison of the two processes.

### Mo-containing materials for fusion reactor components

Due to its robust structural and physical properties, the use of Mo or Mo-alloys (i.e., titanium-zirconium molybdenum (TZM)) in fusion reactors, for example, in the structural components of the reactor such as the divertor or blanket, has been considered. However, because of the potential activation of Mo under the high, fast neutron fluxes when using either D-D or D-T fusion fuels, considerable dose rates from the induced activity and the subsequently generated waste from these materials can be problematic during operation, decommissioning, and in the case of a potential accident^134–136^. Hence, specific materials have been chosen that will reduce the induced radioactivity, mostly those based on carbon (C), silicon (Si), titanium (Ti), iron (Fe), chromium (Cr), and vanadium (V), which include materials such as ferritic-martensitic steels (e.g., EUROFER97, F28H, HT9, etc.), and sintered aluminium. The exchange of Mo for W, its heavier chemical homolog, in component materials has been a particular design strategy to reduce induced radioactivity, although the presence of Mo at limited concentrations is considered acceptable; this has been the basis of reduced activation ferritic-martensitic steels (RAFMs)^137,138^. Alternatively, the use of Mo enriched in specific Mo isotopes that do not significantly activate, i.e., ^96^Mo and ^97^Mo, has been proposed; however, because of enrichment costs, better economic options will need to be developed for commercial applications^139^.

Iida et al. calculated that short-lived radionuclides and their daughters, such as ^91^Mo, ^99^Mo/^99m^Tc, and ^101^Mo/^101^Tc, would contribute to the rapid rise in induced radioactivity during the beginning of reactor operation and throughout its operating lifetime. After two years of operation, the radioactivity of Mo used as a structural material in the blanket of the JAERI tokamak D-T fusion reactor (JFDR) would be nearly two orders of magnitude more than the stainless steel reflector, much of which is attributed to decaying ^99^Mo/^99m^Tc. Seven days post-shutdown, the corresponding dose rate inside of the Mo shield would be around 100 mrem·h^−1^, however, due to adequate thickness of the proposed shield the dose outside would significantly less at about 10 mrem·h^−1^ ^134^. The activation profiles for Mo in ferritic steels such as HT9 were investigated by Youssef and Conn for the SATYR D-D and WITAMIR-I D-T blankets^140^. They showed for the D-T system that ^101^Mo produced in the reactor exhibited a high short-lived level or isotopic radioactive index (IRI_A_). The “softer” spectrum for the D-D system is predicted to enhance (*n*, *γ*) reactions, thus ^101^Mo would have a slightly higher contribution to total activity at shutdown. The activity of ^101^Mo/^101^Tc in the ferritic steel SATYR blanket as a function operating time indicated that, although there is a slight decrease, the activity of the two radioisotopes remains relatively constant throughout the 30 years operation period^141^.

Despite the fact that W is a leading high-Z material for surface components in the divertor and first wall of a fusion reactor, there are concerns about potential performance issues and the strategic uncertainty of relying on one specific material for construction. Brooks et al. surveyed other possible refractory metals, such as Zr, Nb, Mo, Hf, and Ta, and calculated their performance under the ARIES-ACT-1 fusion reactor operating conditions^142^. For example, the U.S. waste disposal rating (WDR) was used to categorise the contribution of these elements after 3.8 MWy·m^−2^ of divertor neutron irradiation, where for a W-based divertor this qualified as low-level waste (WDR < 1 Ci·m^−3^ at 100 y after replacement), while Mo was considered HLW (WDR > 1) even when the armour and divertor were measured together, which reduced the total value. However, the dose rate exposure for remote handling equipment necessary to recycle the divertor are nearly similar for W and Mo after a day or two, in such that recycling for either system could be initiated immediately after shutdown. Similarly to the activity profile, the heat load for Mo (~5 × 10^5 ^W·m^−3^) decreases significantly after one day, which also improves possibilities associated with post-processing^142^. Cepraga et al. presented calculations demonstrating similarities when considering TZM alloy as a divertor material for the Next European Torus (NET)^143^. For TZM (99.369 wt% Mo), both ^101^Mo and ^101^Tc along with ^91^Mo (*t*_1/2_ = 15.49 min) were the first isotopes to decay in less than a day, which also correlated to a diminishment in the total associated radioactivity and decay heat.

### Potential use in nuclear medicine for diagnostics, therapeutics, and/or theranostics

It was not apparent from the literature that ^101^Tc has never been utilised for nuclear medicine purposes in a laboratory or clinical setting. As previously described here in, there is the redundant affiliation of ^101^Tc in nuclear medicine as an impurity generated alongside ^99m^Tc during production. However, there are several accounts that have studied its decay properties in the context as a hypothetical diagnostic, therapeutic, and/or theranostic agent, where the works of our group, i.e., Mausolf et al. and Mayordomo et al., constitute the most detailed explanations to date^15,62^. In these works, the logistical model for fusion-based neutron generator, or an alternative compact accelerator neutron source, production and application of ^101^Tc was compared to the combination of emerging short-lived radiotherapeutic isotopes, such as ^226^Th (*t*_1/2_ = 30.57 min) and ^214^Pb (*t*_1/2_ = 27.06 min), and accelerator-produced PET radioisotopes, such as ^15^O (*t*_*1/2*_ = 122.24 s), ^13^N (*t*_*1/2*_ = 9.97 min), and ^11^C (*t*_1/2_ = 20.36 min), which are generated and distributed in relative proximity to the end-user. Although the use of shorter-lived radioisotopes generally goes against the guidelines concerning the *t*_1/2_ of an ideal therapeutic as outlined by Qaim^144^, where half-lives on the order of several hours to a week are considered the most efficient, it is acknowledged that a part of this justification is associated with the ability to conveniently ship, store, and stockpile these radioisotopes as most are typically produced in a nuclear reactor or require a high-power accelerator isolated an appreciable distance from the patient^145^. However, the possibility of real-time, onsite production using a compact accelerator source, similarly to how radionuclide generators or PET isotopes are utilised, circumvents many of the supply logistics and constraints associated with the use of a short-lived radioisotope.

Furthermore, an assessment of the fundamental decay properties (i.e., *β*^*–*^ and *γ*-ray emissions) of ^101^Tc were compared to the therapeutic properties of ^186^Re (*t*_1/2_ = 3.72 d; *β*_max_ = 1.072 MeV, *I*_β_ = 71.0%), ^188^Re (*t*_1/2_ = 17.01 h; *β*_max_ = 2.120 MeV, *I*_β_ = 70.7%), and ^89^Sr (*t*_1/2_ = 50.563 d; *β*_max_ = 1.495 MeV, *I*_β_ = 70.7%), and the diagnostic ones of ^131^I (*t*_1/2_ = 8.025 d; *E*_γ_ = 364.48 keV, *I*_γ_ = 99.9%) for single-photon emission computed tomography (SPECT) imaging^15^. The clinical application of these isotopes include palliative care, synovectomy, endovascular irradiation, treatment of osseous metastases, neuroendocrine tumours, prostate cancer and thyroid ablation to name a few. Research published by Manjunatha presented dosimetry calculations to determine the effects of various β^–^emitting nuclides in human bone, in particular on parameters regarding yield, intensity, and β^–^ bremsstrahlung dose in human skeletal tissues^146^. The bremsstrahlung dose (Gy·Bq^−1^) as a function of distance from a ^101^Tc source (*β*_max_ = 1.613 MeV) in cortical bone is shown in Supplementary Fig. S10. The calculated bremsstrahlung exposure rates (mR·h^−1^) for ^101^Tc in cortical bone, red marrow, yellow marrow, spongiosa, and cartilage are 78.661, 41.272, 40.642, 55.352, and 49.663 mR·h^−1^, respectively. Other *β-*emitting radionuclides with *β*_*max*_ energies comparable to ^101^Tc that were presented include ^32^P (*t*_1/2_ = 14.268 d; *β*_max_ = 1.710 MeV) and ^89^Sr (Supplementary Fig. S10), both which have historically been utilised in the clinical setting for treating bone diseases^147–149^.

Because of the extensive knowledge-base behind ^99m^Tc diagnostics, it is likely that similar applications and chemistry will be translational for ^101^Tc, although experimental testing will be necessary to demonstrate this. In this regard, ^99m^Tc chemistry related to cold-kits and tagging is well-studied and an array of formulations are produced for routine clinical use. However, limitations related to the time required for synthesis and delivery of the drug will make those which are less time intensive necessary in order to maximize ease of use. For example, pertechnetate [TcO_4_]^–^ is the chemical form eluted from commercial ^99^Mo/^99m^Tc generators, which is both the starting material for successive radiopharmaceutical compounding and an imaging agent in itself. Cold kits that require minimal manipulations and synthesis time (i.e., ≤ 1 *t*_1/2_) which could theoretically be applied for ^101^Tc include but are not limited to: methylene diphosphonate (MDP), hydroxydiphosphonate (HDP), pyrophosphate (PYP), ethylenediamine pentaacetic acid (DTPA), glucoheptonate (GH), N-(2,6-diethylacetanilido) iminodiacetic acid (EHIDA), phytate, sulfur colloid, human serum albumin (HSA) (nano)colloid, HSA microspheres, d,l-hexamethylpropylene amine oxime (d,l-HMPAO), methoxyisobutylisonitrile (MIBI), and tetrofosmin^150^. New kit formulations specifically designed for ^101^Tc, or the development of rapid, automated techniques that streamline compounding, such as microwave-assisted syntheses or microfluidic chip systems, could provide alternative solutions^151,152^. Similarly, there are drug delivery routes, such as Technegas® (e.g., inhalation) and the use of catheters (e.g., intra-arterial or intracavitary infusion brachytherapy, convection-enhanced delivery (CED), etc.) that allow for the accelerated introduction of radiopharmaceuticals to points of critical concern, which could be leveraged for this shorter-lived isotope^153,154^.

## Conclusion and outlook

From the shadows of its isotopic brethren ^99m^Tc and ^99g^Tc, the short-lived ^101^Tc emerges as an equally interesting and potentially useful isotope. The known literature summarised here showcases its array of unique nuclear properties, production and isolation routes, and applications in various areas of research and industry. Improved understanding of its fundamental nuclear structure as a heavier, neutron-rich Tc isotope gives further clarity to the relationship amongst all Tc isotopes and insight into the inherent radioactive nature of Tc. The straightforward production of ^101^Tc through (*n*, *γ*) reactions on natural or enriched ^100^Mo-containing samples, even with lower yielding neutron fluxes, is quite promising considering ongoing advances in compact accelerator neutron sources (CANS) technology. Meanwhile, its shorter *t*_1/2_ makes it manageable to work with and its stable isobar end-member ^101^Ru negates the need for radioactive waste management or the possibility of long-term contamination scenarios. As the element Tc, an array of oxidation states and rich coordination chemistry are accessible, and it will be interesting to see what similar chemistries for other Tc isotopes will be applicable for ^101^Tc.

A better understanding of the potential of fission-derived and neutron-activated ^101^Tc, either paired together or as unique signatures, for applications related to on- or offline nuclear fuel monitoring may play a role in determining fuel composition, enrichment, burnup, etc., and may aid in the context of nuclear proliferation and materials interrogation that has only been previously touched upon. This function will be particularly important with the geopolitical and technological shift from HEU-fueled reactors to LEU ones, where high-density U-Mo compositions have become of primary interest. There are also implications for improvements in neutron economy and the potential for reducing radiotoxicity and/or long-lived waste generation in primary and ancillary nuclear components depending on the Mo isotopics. With the recent efforts and focus on the development and deployment of commercial fusion energy, it will be key to utilise materials that are not only structurally and functionally robust, but will also minimise problematic radioactive wastes; thus, tailoring Mo isotopics could be beneficial where short-lived radioactive isotopes, e.g., ^101^Mo/^101^Tc, or neutron poisons, e.g., ^95^Mo, are dominant.

Stable isotope enrichment is another area of scientific, commercial, and industrial interest. The large-scale use of stable isotopes, however, is significantly impeded by the small number of enrichment facilities worldwide and the lack of cost/resource-effective processes that can be employed, especially for bulk quantities. As discussed in this review, enrichment of particular Mo isotopes fractions, i.e., ^98^Mo and ^100^Mo, is crucial for some applications, such as radioisotope production, and would greatly benefit others where sourcing appreciable amounts is not quite yet an option, i.e., nuclear fuels and materials. However, not discussed but relevant to the topic of isotope enrichment, is the potential for generating an isotopically pure stream of ^101^Ru from ^101^Tc. When using a Mo target and a neutron source, the Mo (natural or enriched in ^100^Mo) starting material is simultaneously transmuted and upgraded from a minor metal of relative fiscal value through ^101^Mo/^101^Tc to Ru, a platinum group metal (PGM) worth several orders of magnitude more. Mining PGMs, such as Ru, from spent nuclear fuel has been proposed^155^; whether appreciable amounts of Ru could be generated through non-fission-based means and whether it would be financially worthwhile has yet to be determined.

Within the field of nuclear medicine, the complexities related to just-in-time production and distribution of radioisotopes, especially ones that require unique infrastructure like a high-flux nuclear reactor or high-energy accelerator, make logistics of production and supply a challenge^156,157^. The authors believe that providing an alternate pipeline of medical radioisotopes that can be produced in a distributed fashion closer to the point of use may help to alleviate and possibly avoid some supply chain issues. ^101^Tc may be a likely candidate for this model, however, significant research and clinical testing will first be necessary to determine its effectiveness and whether there is potential for it as a medical radioisotope.

Although many pathways for its production have been established, the knowledge of isolation and separation methodologies as well as known applications for ^101^Tc are limited. In general, the gaps in the known literature concerning ^101^Tc, for example, in comparison to its Tc isotope brethren ^99m^Tc and ^99g^Tc, highlight the apparent need for further investigative work in order to better elucidate trends in nuclear and chemical behaviour across the range of Tc isotopes. The authors hope that this will be the first of many reviews on this unique and interesting Tc isotope.

## Supplementary information

Johnstone_PR File

Supplementary Material

## Data Availability

Data is contained within the article and Supplementary Information.
