# Supplementary material for: Discovery, nuclear properties, synthesis and applications of technetium-101
Source: Commun Chem. 2022 Oct 20;5:131. doi: 10.1038/s42004-022-00746-9 (PMC9814870; doi:10.1038/s42004-022-00746-9)
Supplement: Supplementary file 2 — Supplementary Material [file 42004_2022_746_MOESM2_ESM.pdf]

Supplementary Information

Discovery, nuclear properties, synthesis and applications of technetium-101

E. V. Johnstone,[1,*] *N. Mayordomo,*[2] *and E. J. Mausolf*[1]

[1] Innovative Fuel Solutions (IFS) LLC., North Las Vegas, NV, 89031 USA

[2] Helmholtz-Zentrum Dresden Rossendorf (HZDR), Institute of Resource Ecology, Bautzner Landstraße 400, 01328 Dresden, Germany

Email: Erikvjohnstone@gmail.com

Supplementary Figures and Tables

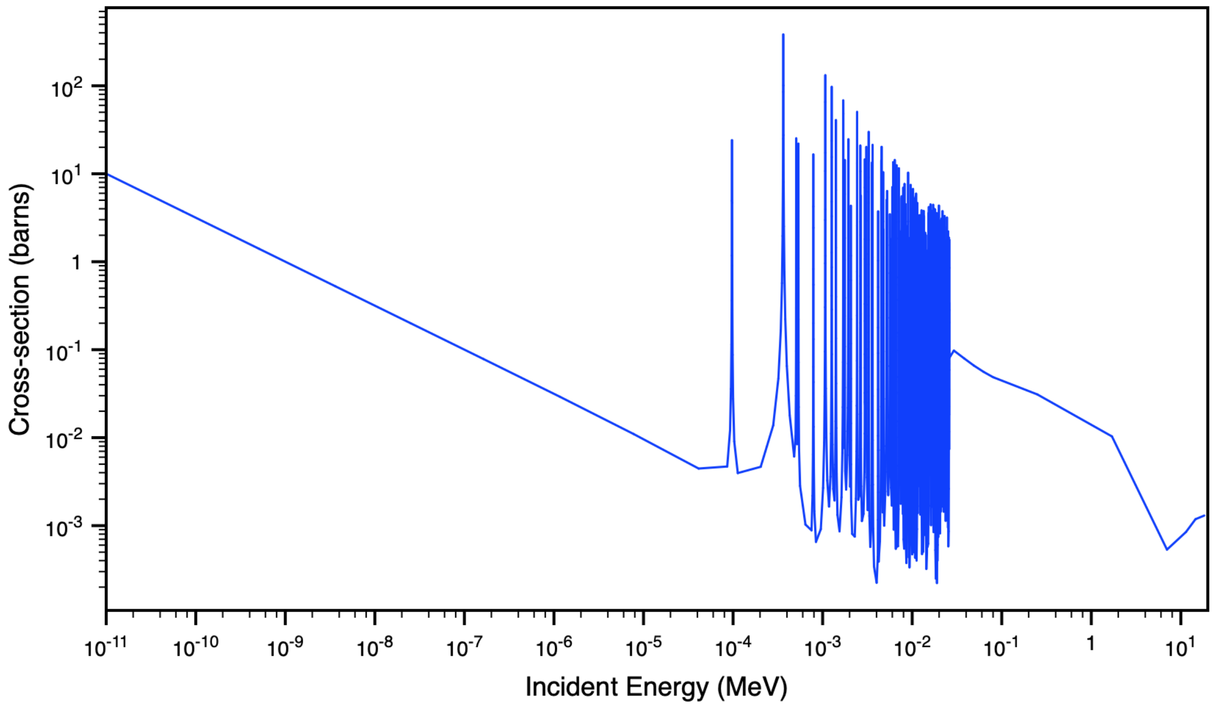

Supplementary Figure S1. ENDF/B-VIII calculated excitation function of the ^{100}Mo(n,γ)^{101}Mo reaction.

Supplementary Table S1. Transitions in ^{101}Ru from the decay of ^{101}Tc based on the Evaluated Nuclear Structure Data File (ENDSF) database [1].

Eγ [keV]	Iγ [keV]	$J_i^\pi \rightarrow J_f^\pi$	Classification [keV]	Multipolarity	δ
127.22 *3*	29.6 *9*	$3/2^+ \rightarrow 5/2^+$	127.22 → 0	M1+E2	+0.17 *4*
179.60 *4*	6.5 *5*	$7/2^+ \rightarrow 3/2^+$	306.83 → 127.22	E2	—
182.12 *5*	18.0 *6*	$5/2^+ \rightarrow 3/2^+$	311.35 → 127.22	M1	—
233.70 *5*	3.00 *13*	$7/2^+ \rightarrow 5/2^+$	545.06 → 311.05	M1(+E2)	—
238.25 *5*	3.38 *14*	$7/2^+ \rightarrow 7/2^+$	545.06 → 306.83	M1(+E2)	—
281.6 *7*	0.30 *6*	$11/2^+ \rightarrow 9/2^+$	1001.16 → 720.02	—	—
295.17 *13*	0.55 *9*	$3/2^+ \rightarrow 3/2^+$	422.48 → 127.22	—	—
306.83 *3*	1000 *50*	$7/2^+ \rightarrow 5/2^+$	306.83 → 0.0	M1+E2	-0.10 *5*
311.28 *8*	2.36 *25*	$5/2^+ \rightarrow 5/2^+$	311.35 → 0.0	(M1)	—
322.01 *4*	0.41 *5*	$(7/2)^+ \rightarrow 3/2^+,5/2^+$	938.47 → 616.37	—	—
383.83 *10*	0.32 *7*	$9/2^+ \rightarrow 7/2^+$	928.72 → 545.06	—	—
393.30 *8*	1.13 *9*	$(7/2)^+ \rightarrow 7/2^+$	938.47 → 545.06	—	—
422.02 *16*	0.36 *5*	$3/2^+ \rightarrow 5/2^+$	422.48 → 0.0	—	—
489.10 *15*	0.37 *5*	$3/2^+,5/2^+ \rightarrow 3/2^+$	616.37 →127.22	—	—
516.13 *8*	1.11 *8*	$(7/2)^+ \rightarrow 3/2^+$	938.47 → 422.48	—	—
531.42 *5*	11.3 *4*	$(7/2)^+ \rightarrow 5/2^+$	842.79 → 311.35	—	—
545.05 *6*	67.2 *18*	$7/2^+ \rightarrow 5/2+$	545.06 → 0.0	M1+E2	-0.98 *10*
*616.3	0.17 *4*	—	—	—	—
617.31 *9*	0.60 *4*	$9/2^+ \rightarrow 5/2^+$	928.72 → 311.35	—	—
621.99 *12*	0.93 *6*	$9/2^+ \rightarrow 7/2^+$	928.72 → 306.83	—	—
627.00 *6*	4.9 *2*	$(7/2)^+ \rightarrow 5/2^+$	938.47 → 311.35	—	—
631.74 *12*	0.45 *3*	$(7/2)^+ \rightarrow 7/2^+$	938.47 → 306.83	—	—
*673.4 *6*	0.35 *5*	—	—	—	—
694.30 *15*	0.61 *7*	$11/2^+ \rightarrow 7/2^+$	1001.16 → 306.83	—	—
715.53 *4*	7.6 *3*	$(7/2)+ \rightarrow 3/2^+$	842.79 → 127.22	—	—
720.02 *5*	2.42 *12*	$9/2^+ \rightarrow 5/2^+$	720.02 → 0.0	—	—
811.13 *9*	0.65 *6*	$(7/2)^+ \rightarrow 3/2^+$	938.47 → 127.22	—	—
842.73 *7*	2.53 *10*	$(7/2)^+ \rightarrow 5/2^+$	842.79 → 0.0	—	—
*911.57 *12*	0.6 *2*	—	—	—	—
928.72 *6*	1.25 *8*	$9/2^+ \rightarrow 5/2^+$	928.72 → 0.0	—	—
938.65 *20*	0.93 *6*	$(7/2)^+ \rightarrow 5/2^+$	938.47 → 0.0	—	—

** γ-ray not assigned in level scheme*

**italicised numbers represent associated errors

Supplementary Table S2. Experimental data for production of ^{101}Tc via the (n,^{3}He) reaction on ^{103}Rh.

Year	$E_{neutron}$ (MeV)	σ (nb)	Ref.
1965	14.8	13 ± 6.5	Frevert [2]
1966	14.7	0.4	Gray [3]
1966	14.7	1.2 ± 0.6	Csikai [4]
1968	14.8	2.0 ± 0.6	Husain [5]
1974	14.6	16 ± 7	Diksic [6]

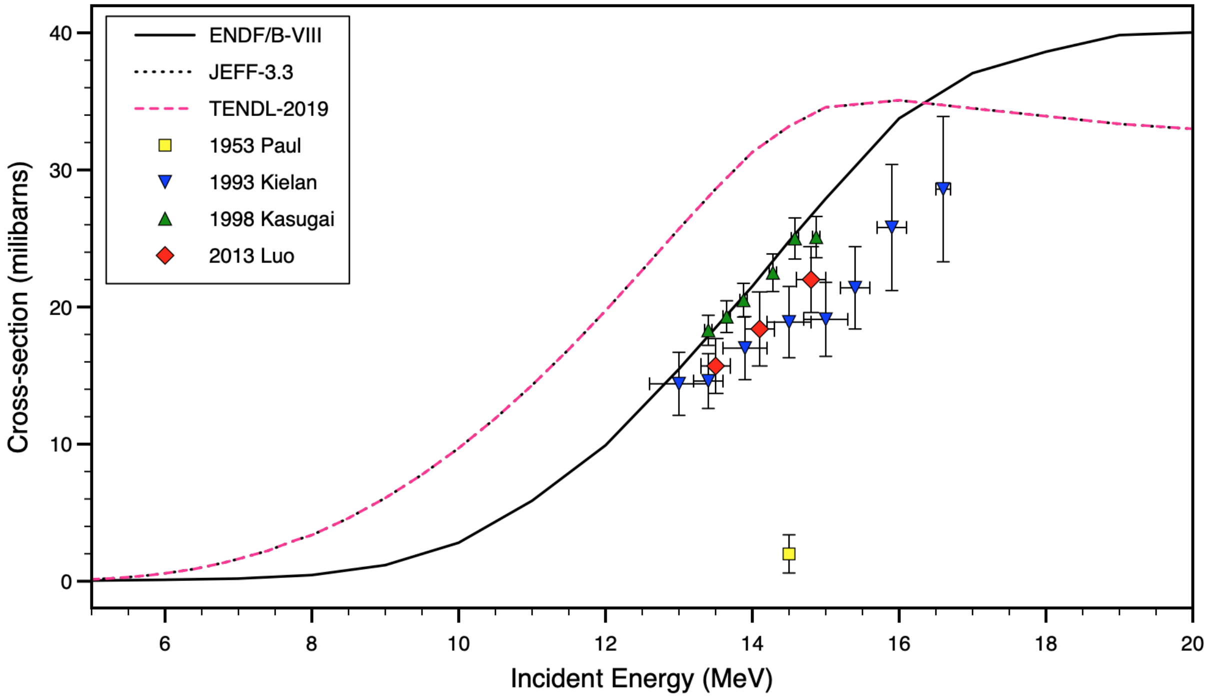

Supplementary Figure S2. Excitation function of the ^{101}Ru(n,p)^{101}Tc reaction. Plotted symbols represent the experimental data and the lines represent modelled data. Error bars are included.

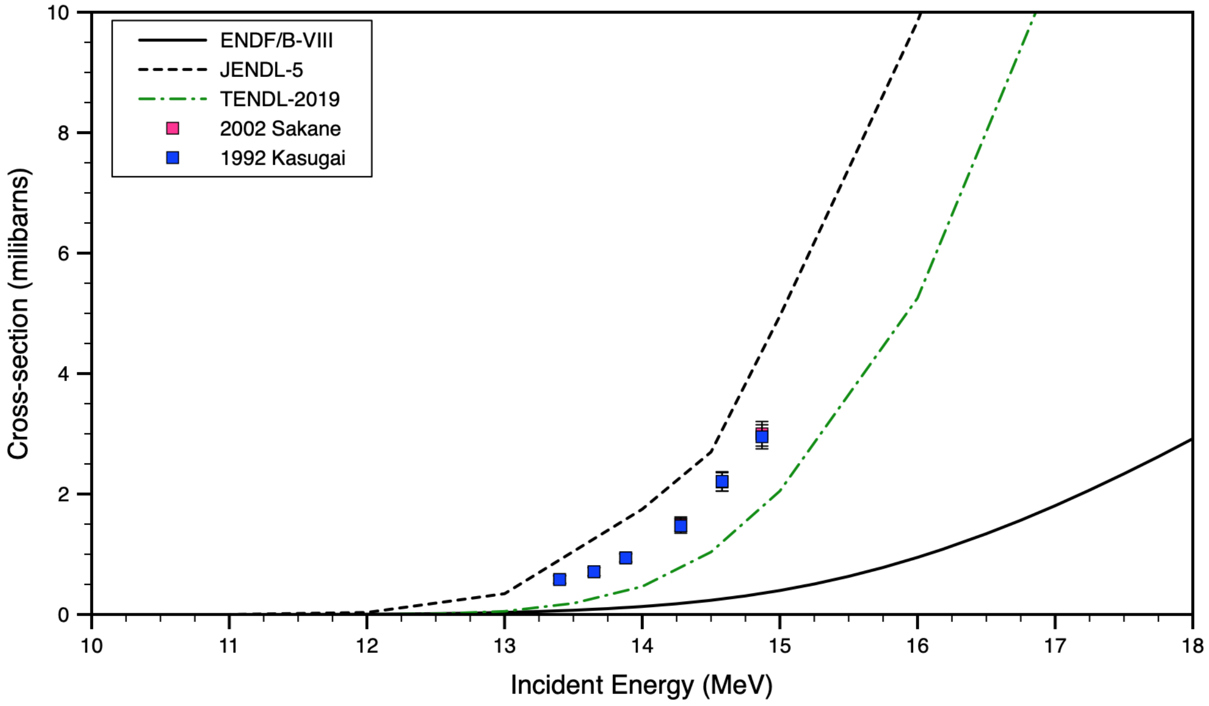

Supplementary Figure S3. Excitation function of the ^{102}Ru(n,d+np) ^{101}Tc reaction. Plotted symbols represent the experimental data and the lines represent modelled data. Error bars are included.

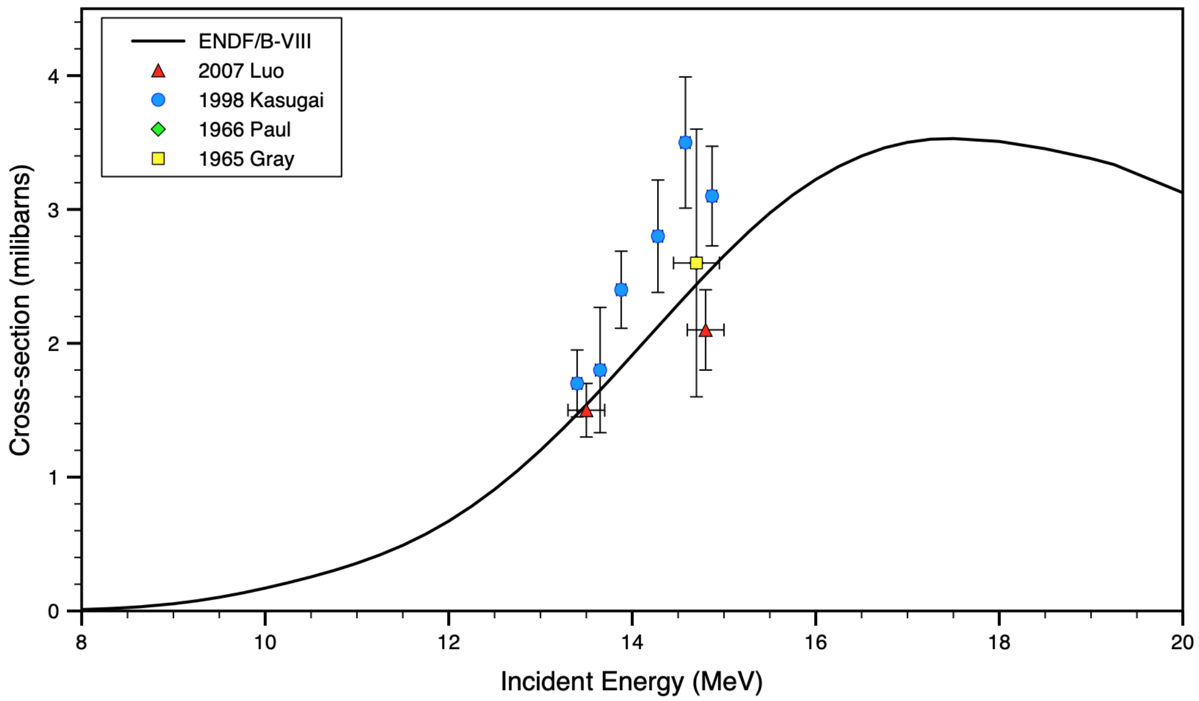

Supplementary Figure S4. Excitation function of the ^{104}Ru(n,α)^{101}Mo via the reaction. It is noted that the data points for Paul and Gray overlap. Plotted symbols represent the experimental data and the line represents modelled data. Error bars are included.

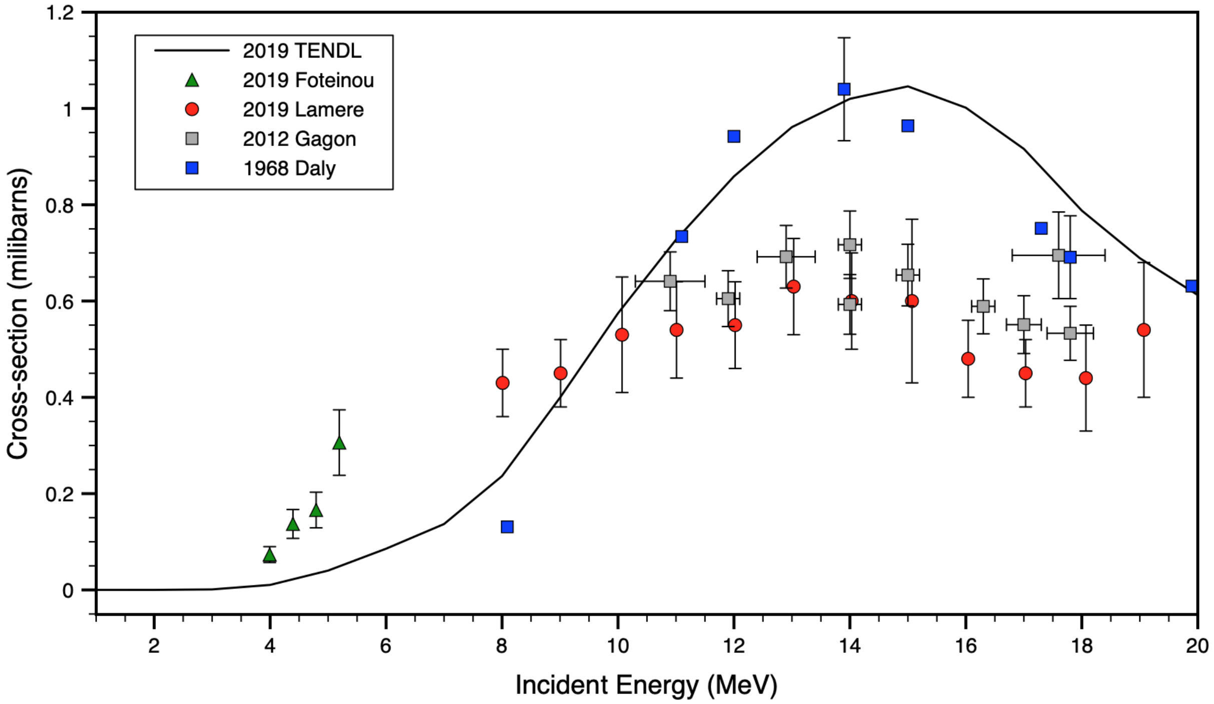

Supplementary Figure S5. Excitation function of the $^{100}Mo(p,\gamma)^{101}Tc$ reaction. Plotted symbols represent the experimental data and the lines represent modelled data. Error bars are included.

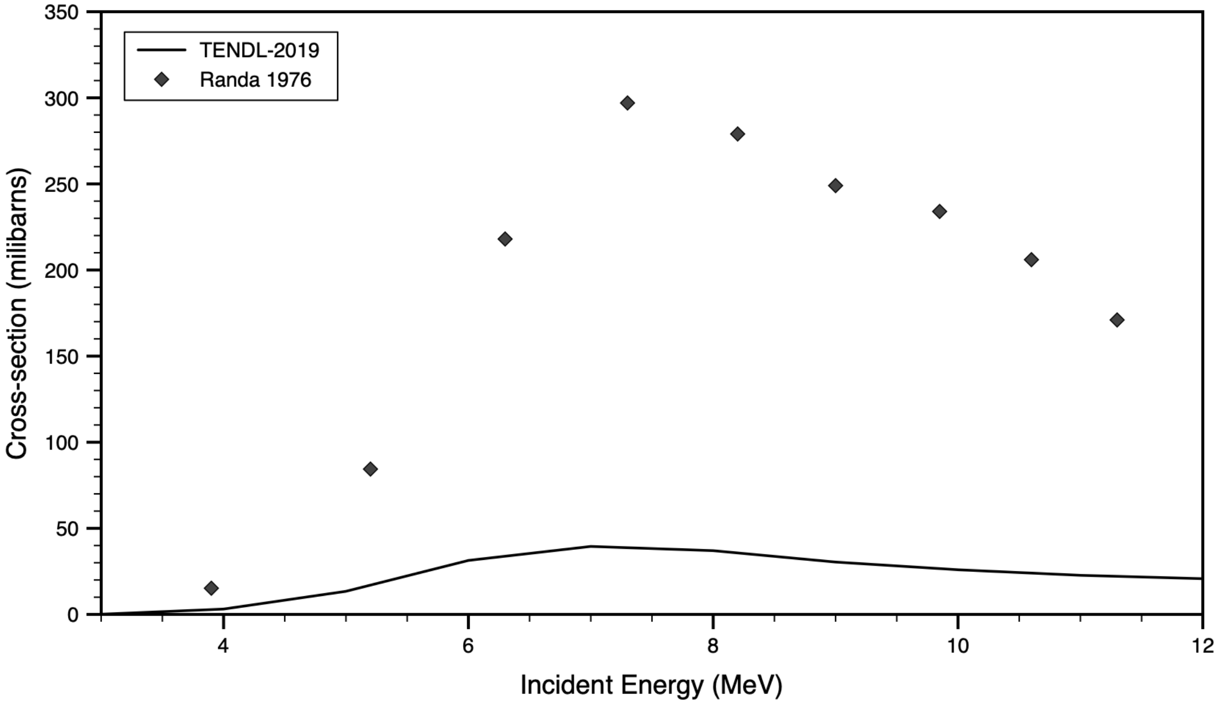

Supplementary Figure S6. Excitation function of the $^{100}Mo(d,n)^{101}Tc$ reaction. Plotted symbols represent the experimental data and the line represents modelled data. Error bars are included.

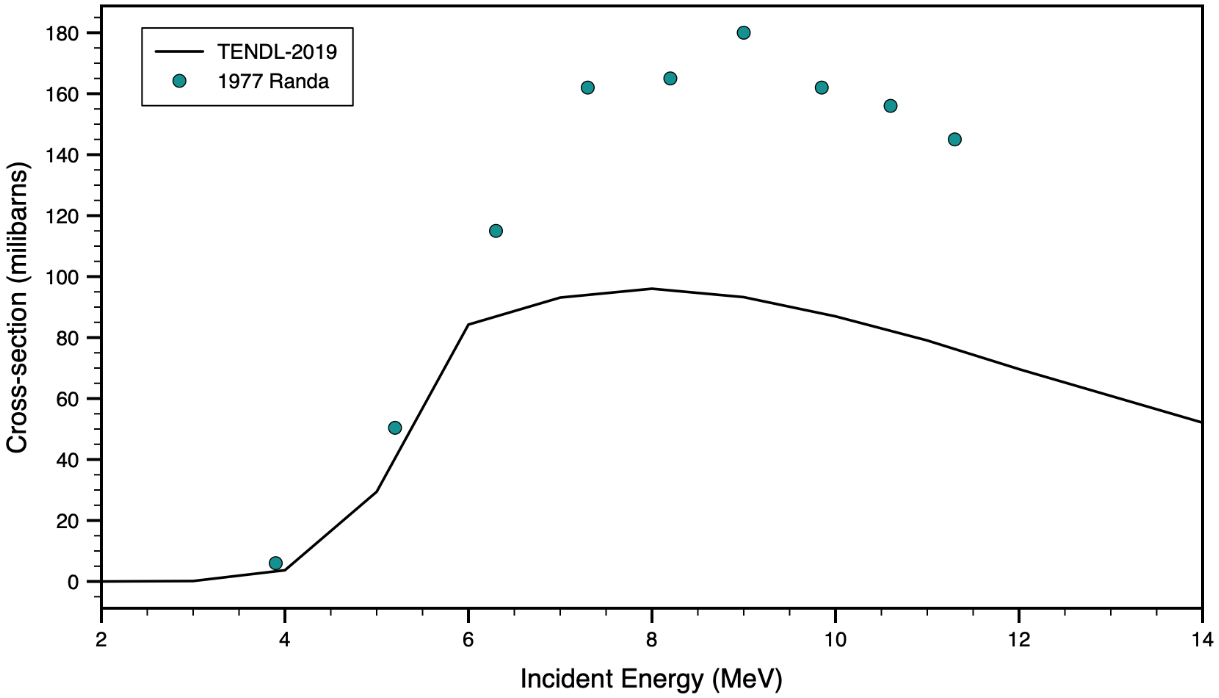

Supplementary Figure S7. Excitation function of the $^{100}Mo(d,p)^{101}Mo$ reaction. Plotted symbols represent the experimental data and the line represents modelled data. Error bars are included.

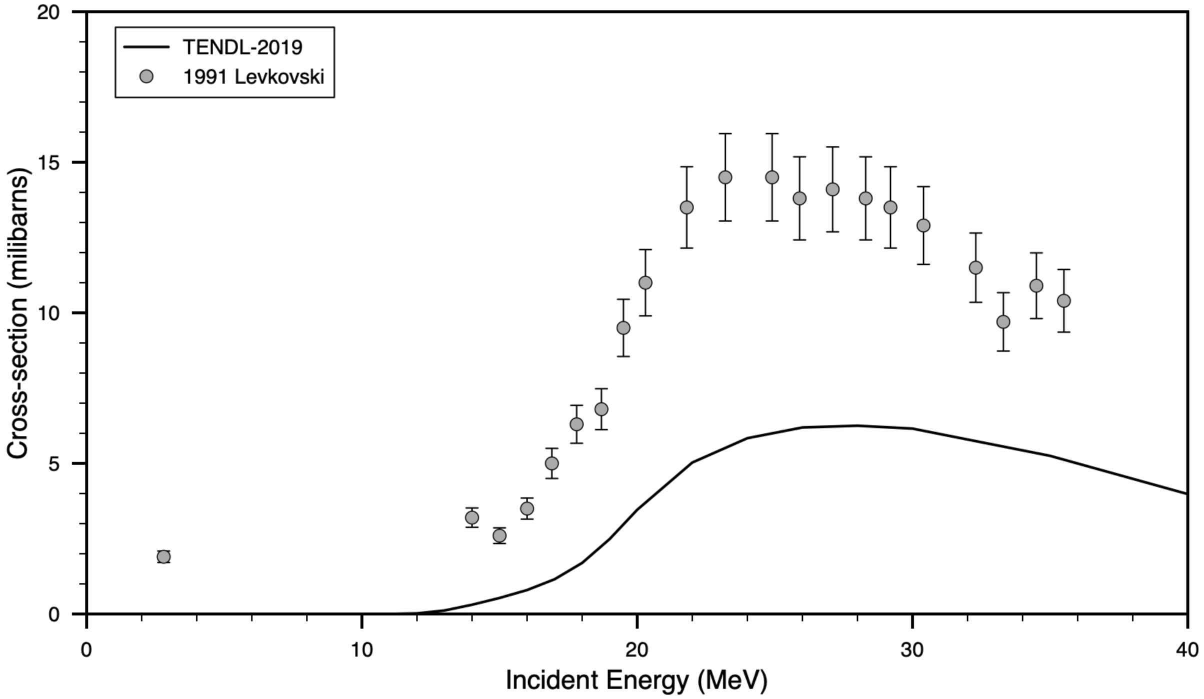

Supplementary Figure S8. Excitation function of the $^{98}Mo(\alpha,p)^{101}Tc$ reaction. Plotted symbols represent experimental data and the line represents modelled data. Error bars are included.

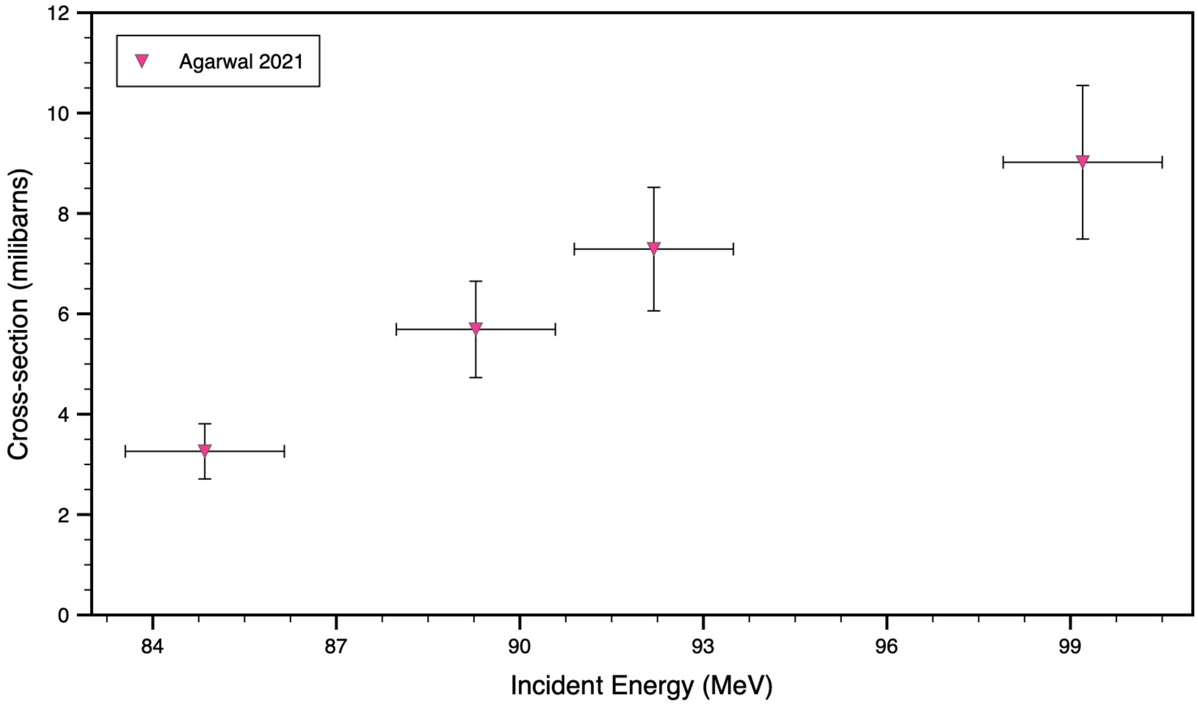

Supplementary Figure S9. Excitation function of the $^{93}Nb(^{18}O,2\alpha 2p)^{101}Tc$ reaction. Error bars are included.

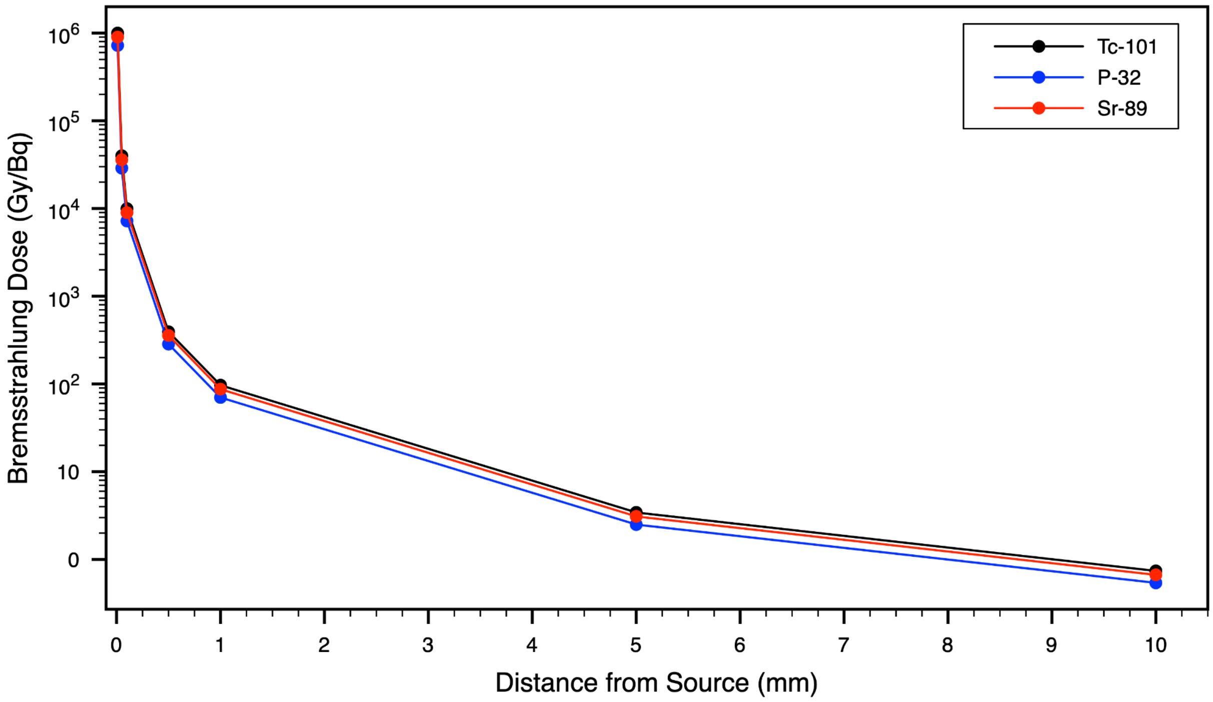

Supplementary Figure S10. Calculated bremsstrahlung doses in cortical bone as a function of distance (mm) for ^{101}Tc (black), ^{32}P (blue), and ^{89}Sr (red) [7].

Supplementary References

1. Plots produced using the code ENSDAT, written by R.R. Kinsey, National Nuclear Data Center, Brookhaven National Laboratory, Upton, NY, U.S.A.

2. Fervert, E. (*n*,^{3}He) cross-section measurements on ^{55}Mn, ^{59}Co, ^{75}As and ^{103}Rh for 14.8 neutrons. *Acta. Phys. Austriaca.* **100**, 202 (1965).

3. Gray, P. R., Zander, A. R., Ebery, T. G. Activation cross sections for reactions of Rh and Ru with 14.7 MeV neutrons. *Nuc. Phys.* **75**, 215–225 (1966).

4. Csikai, J. Investigation of ^{103}Rh(*n*,^{3}He)^{101}Tc reaction. *Act. Phys. Aca. Sci. Hungar.* **21**, 229–233 (1966).

5. Husain, L., Bari, A., Kuroda, P. K. 14.8 MeV Neutron cross-section for (*n*,^{3}He) and other rare nuclear reactions on ^{103}Rh. *J. Inorg. Nucl. Chem.* **30**, 3145–3149 (1968).

6. Diksic, M., Strohal, P., Slaus, I. (*n*,^{3}He) and (*n*,*t*) reaction cross-sections at 14 MeV. *J. Inorg. Nucl. Chem.* **36**, 477–485 (1974).

7. Manjunatha, H. C. A dosimetric study of Beta induced bremsstrahlung in bone. *Appl. Rad. Iso.* **94**, 282–293 (2014).
