# Supplementary material for: Discovery, nuclear properties, synthesis and applications of technetium-101
Source: Commun Chem. 2022 Oct 20;5:131. doi: 10.1038/s42004-022-00746-9 (PMC9814870; doi:10.1038/s42004-022-00746-9)
Supplement: Supplementary file 1 — Johnstone_PR File [file 42004_2022_746_MOESM1_ESM.pdf]

nature portfolio

Peer Review File

Discovery, nuclear properties, synthesis and applications of technetium-101

Open Access This file is licensed under a Creative Commons Attribution 4.0 International License, which permits use, sharing, adaptation, distribution and reproduction in any medium or format, as long as you give appropriate credit to the original author(s) and the source, provide a link to the Creative Commons license, and indicate if changes were made. In the cases where the authors are anonymous, such as is the case for the reports of anonymous peer reviewers, author attribution should be to 'Anonymous Referee' followed by a clear attribution to the source work. The images or other third party material in this file are included in the article's Creative Commons license, unless indicated otherwise in a credit line to the material. If material is not included in the article's Creative Commons license and your intended use is not permitted by statutory regulation or exceeds the permitted use, you will need to obtain permission directly from the copyright holder. To view a copy of this license, visit http://creativecommons.org/licenses/by/4.0/.

REVIEWERS' COMMENTS:

Reviewer #1 (Remarks to the Author):

The authors summarize the discovery history, physical and chemical properties, production and separation methods, and practical applications of Tc-101 in this review. The authors have a solid research foundation in this field and give a perfect review about this area. I strongly recommend it publishing in this journal after addressing some minor issues:
1. Authors should unify the representation of isotopes in the full text: such as 99gTc and Technetium-99g. It is indicated as Technetium-99g (99gTc) for the first time and referred to 99gTc later.
2. Table 3 occupies too much space and can be adjusted into the Supporting Information.
3. Authors should adjust the insertion of special symbols so that the content is fully displayed in the pdf version. For example: line 192 "2×1010 n□s-1", line 306 "100Mo(□,p)101Tc", Line 374 "(C□H□)-AsCl".
4. The authors should add a comparison and summary of the various methods after discussing the separation methods of Tc-101.
5. The authors believe that Tc-101 could be a medical radioisotope with potential application value. So the authors should select specific cases for discussion in Section 6.4.
6. Conclusion and Future Perspectives should be expanded appropriately. The authors should highlight the special advantages of Tc-101 in a certain range of applications, thereby highlighting the importance of research in this field.

Reviewer #2 (Remarks to the Author):

Technetium-99m is the work-horse of diagnostic nuclear medicine. Several times in the past, delivery of sufficient amounts of Tc-99m in the form of the Mo-99/Tc-99m generators were jeopardized due to shut downs of reactors or interruption due to maintenance. During these periods, the interest in other isotopes of Tc was coming up and Tc-94 (PET) was of particular interest. As the authors emphasize, Tc-101 has the properties for clinical on-site applications but in depth studies have not or rarely been published. Tc-101 is clearly not in the focus of the nuclear medicine community, but has been applied to other technical or fundamental purposes as comprehensively outlined in section 6. This review is clearly written by experts, its scientific quality is impressive and the content very well organized. As far as I can see, the relevant publications are quoted in a proper context and with balanced critics. From the scientific quality, the manuscript matches the high requirements of "Communications Chemistry". However, there is no or very little chemistry in it. The manuscript describes essentially physical properties and nuclear physics of Tc-101. Eventually, the "chemical probes" focus of Commun. Chem. is closest to the ms content's, but "physical probes" would describe the ms probably better. Regarding the perspective of Tc-101, easily available and cheap Tc-99m is strongly competed by PET radionuclides and I have my strong doubts that Tc-101 will ever be seriously considered by the community, although its chemistry is equal to the one of Tc-99m. The authors claim theranostics as a perspective and potential field, however, the half-life time is much too short for any therapeutic application, albeit the beta energies are fine.
In conclusion, the quality of this review is very high (no concerns) but it is too specialized and does eventually not match the interests of Commun. Chem. readers. In this sense I leave the decision to the editor.

Responses to Referees

Discovery, nuclear properties, synthesis and applications of technetium-101

Erik V. Johnstone,[1,] Natalia Mayordomo,[2] and Edward. J. Mausolf[1]*

Email: Erikvjohnstone@gmail.com

Reviewer 1:

1. Authors should unify the representation of isotopes in the full text: such as ^{99g}Tc and Technetium-99g. It is indicated as Technetium-99g (^{99g}Tc) for the first time and referred to ^{99g}Tc later.

The authors acknowledge that use of "Technetium-99g" was inconsistent and has been replaced with "^{99g}Tc" to maintain consistent notation throughout the manuscript. However, to provide clarification for the reader the notation form of "Technetium-101" is used in the manuscript title and in the abstract where it is first mentioned along with the corresponding "^{101}Tc" shorthand notation. The shorthand notation is employed throughout the rest of the manuscript.

2. Table 3 occupies too much space and can be adjusted into the Supporting Information.

The authors agree with the Reviewer and have relocated "Table 3" from the manuscript into the Supplementary Information where it is now "Table S1"; other corresponding tables in the manuscript and SI have been appropriately relabelled to adjust for this action.

3. Authors should adjust the insertion of special symbols so that the content is fully displayed in the pdf version. For example: line 192 "2×1010 n□s-1", line 306 "100Mo(□,p)101Tc", Line 374 "(C□H□)-AsCl".

The authors acknowledge these errors, likely a result of compatibility issues when using different document processing software, and have ensured that they have been corrected.

4. The authors should add a comparison and summary of the various methods after discussing the separation methods of Tc-101.

The authors recognize how a comparison and summary portion for this section would be valuable to the reader and consistent with the focus of the journal, especially considering that it is heavily oriented towards the chemical processing techniques and fundamental chemistry of the different elemental systems being investigated. Thus, a comparison and summary has been added at the end of the "5. Separation" section. In doing so, the authors have also tried to briefly tie in the relationship of known Tc chemistry associated with fission-based processes and those aimed at the development of ^{99m}Tc, as the both of these areas have been thoroughly explored, and it is likely there will be opportunities for similar chemistries can be applied for ^{101}Tc.

5. The authors believe that Tc-101 could be a medical radioisotope with potential application value. So the authors should select specific cases for discussion in Section 6.4.

The authors concede that it is not very clear what the Reviewer means by "specific cases for discussion." Admittedly, the authors realize that this section under "Applications" is relatively underwhelming and very little experimental work has been performed in the way of proving ^{101}Tc to be a medical isotope; the works cited are calculation-based / theoretical at this point in time, although they are pinpointed towards this subject matter. This being said, the authors have made a point to state that this is a "potential use" of the radioisotope, and the listed references provide calculations and comparative arguments that support this possibility. For example, understanding the inherent decay properties of ^{101}Tc, such as its relative β_{max} and gamma decay energies in comparison to known radiodiagnostic and therapeutic isotopes, such as ^{32}P, ^{89}Sr, ^{186}Re, ^{188}Re, and ^{131}I. Fundamentally speaking, ^{101}Tc exhibits comparable nuclear behavior with the exception of its half-life. However, it is not unusual to use short(er)-lived radioisotopes in the field of nuclear medicine, such as PET isotopes for diagnostics as well as some

short(er)-lived therapeutic ones, i.e., ^{226}Th, ^{214}Pb, and ^{213}Bi. The major impediment behind the use of these radioisotopes is generally associated with the ability to: 1) produce / store / stockpile, 2) process, and 3) deliver to the patient. The authors have further elaborated on the possibilities they foresee with parallels made to current onsite-produced radioisotopes, ^{99m}Tc chemistry and drug delivery routes that would potentially make ^{101}Tc a viable medical radioisotope.

Finally, this is a first-of-its-kind review on ^{101}Tc and the authors hope that it will bring newfound interest in the radionuclide. It is not unusual for a single subject to have multiple reviews written over time. As the first one, the authors believe that by pointing out gaps or deficiencies (a.k.a., opportunities) in regard to the subject matter as a whole is valuable. If the additions / edits made to the manuscript have not addressed what the Reviewer has requested, the authors will be glad to adjust the content appropriately in order to do so.

6. Conclusion and Future Perspectives should be expanded appropriately. The authors should highlight the special advantages of Tc-101 in a certain range of applications, thereby highlighting the importance of research in this field.

The authors agree with the Reviewer. The “Conclusion and Future Perspectives” has been expanded upon with the intent of elaborating on the special advantages and importance of the field of ^{101}Tc research.

<u>Reviewer 2:</u>

From the scientific quality, the manuscript matches the high requirements of "Communications Chemistry". However, there is no or very little chemistry in it. The manuscript describes essentially physical properties and nuclear physics of Tc-101. Eventually, the "chemical probes" focus of Commun. Chem. is closest to the ms content's, but "physical probes" would describe the ms probably better. Regarding the perspective of Tc-101, easily available and cheap Tc-99m is strongly competed by PET radionuclides and I have my strong doubts that Tc-101 will ever be seriously considered by the community, although its chemistry is equal to the one of Tc-99m. The authors claim theranostics as a prospective and potential field, however, the half-life time is much too short for any therapeutic application, albeit the beta energies are fine.
In conclusion, the quality of this review is very high (no concerns) but it is too specialized and does eventually not match the interests of Commun. Chem. readers. In this sense I leave the decision to the editor.

The authors appreciate the Reviewer's insight and perspective on the manuscript. The authors concur that the manuscript has a much broader scope of ^{101}Tc, where the chemistry pertaining to it is only a piece of the overall story. That said, the authors believe the readers will realize that in comparison to other Tc isotopes like ^{99m}Tc and ^{99g}Tc (and a few others) the extent of reported chemical studies on ^{101}Tc is limited, and thusly why it also does not constitute a large section of the manuscript. Admittedly, the authors also were slightly concerned that this might not be sufficient enough to satisfy the scope of the journal, but they believe it will find an appropriate audience and interest amongst the journal’s reader-base.

As far as the topic of ^{101}Tc as a potential theranostic, the authors have given this idea much thought and consideration. The authors agree that it lies outside the typical definition of what constitutes most radiotherapeutic isotopes, however, not significantly enough to be disregarded. For example, much of the hindrance behind the use of short-lived isotopes is related to production, stockpiling / supply, and delivery to the patient. The authors have added further discussion to the manuscript where these details are discussed and some potential, straightforward solutions are provided. Furthermore, use of a shorter-lived radiotherapeutic provides different opportunities than the current fleet being commercially and clinically employed, which mostly exhibit half-lives from several hours up to a week or longer. Theoretically, a shorter-lived radioisotope like ^{101}Tc would allow for more treatments and provide the possibility for adjusting radiation dose along the course of treatment. For example, radiotherapy treatments using an external beam linac photon source occur 5-days a week and span anywhere from 4 to 8-weeks in total for a patient [1,2]; the administered dose can be adjusted as appropriate depending on the schedule and patient’s progress. A shorter-lived radiotherapeutic isotope also circumvents situations, such as 1) long-term, extraneous dose to patient / healthy organs, tissues, and cells, 2) having to isolate the patient for long periods due to potential extraneous radioactive dose to the public [3], 3) radioactive materials accountability [4], and 4) challenges associated with nuclear waste accumulation / storage / contamination scenarios.

As discussed in the manuscript, one of the biggest issues with current radiotherapeutic nuclides is sourcing them. Long irradiation times in nuclear reactors / high power accelerators or even special nuclear material is often

required for their production - this pertains to ^{177}Lu, ^{225}Ac, ^{188}Re, ^{90}Y and ^{131}I amongst others. An isotope like ^{101}Tc would require some special infrastructure, however, not so drastically different than what is necessary for a biomedical cyclotron or linac, both of which already generate (secondary, fast) neutrons, in a hospital or medical facility. The shorter half-lives of ^{101}Mo and ^{101}Tc allow for near-saturation in production within a short duration (~1 h), which would allow for fast ramp up of production and multiple production runs throughout the day. Depending on the strength of the neutron source, size of the target, and processing time required, ^{101}Tc will persist due to the ^{101}Mo-^{101}Tc couple for several hours after end of bombardment (EOB). For example, in TRIGA reactors (~$1x10^{12}$ n/s·cm^2) a cooling time of anywhere from 3 to 6-hours is recommended to ensure that all ^{101}Mo / ^{101}Tc has decayed prior to processing irradiated Mo targets for the purpose of low specific activity ^{99}Mo / ^{99m}Tc [5,6].

Lastly, the costs of current targeted radionuclide therapies can range upwards of ~$10,000 to ~$50,000 per dose with multiple doses typically required (i.e., 3 to 6 depending on the procedure) totaling anywhere between ~$100,000 to ~$250,000 per patient [7–9]. In comparison, external beam radiotherapy and brachytherapy for cervical cancer cost approximately ~$9,000 and ~$13,000, respectively [10], and treatment of prostate cancer ranged from ~$2,700 to ~$10,000 depending on the technique(s) employed [11]. Obviously, these prices can vary and fluctuate depending on location / country, timing, etc., but in general they are an order of magnitude less in present day costs than those reported for emerging targeted radionuclide therapies. The potential of using lower-costing accelerator neutron sources [12] in conjunction with previously existing Tc kit chemistry could provide possible avenues for reducing overall costs for comparable therapy.

Although the constraints imposed by the half-life of ^{101}Tc will be challenging, the authors believe there is still sufficient reason to pursue it as a medical isotope given the potential benefits of the system. The authors do concede that the necessary laboratory and clinical testing must be performed before any guarantee of its effectiveness can be made with certainty.

References

1. American Cancer Society. Radiation for Breast Cancer (accessed 12 Sep. 2022) https://www.cancer.org/cancer/breast-cancer/treatment/radiation-for-breast-cancer.html
2. Prostate Cancer UK. External Beam Radiotherapy (accessed 12 Sep. 2022) https://prostatecanceruk.org/prostate-information/treatments/external-beam-radiotherapy
3. SNMMI. Fact Sheet: Guidelines for Patients Receiving Radioiodine I-131 Treatment (accessed 15 Sep 2022). https://www.snmmi.org/AboutSNMMI/Content.aspx?ItemNumber=5609
4. Nathan, Y. Y., Rule, W. G., Sio, T. T., Ashman, J. B., & Nelson, K. L. Radiation contamination following cremation of a deceased patient treated with a radiopharmaceutical. *JAMA*, **321(8)**, 803–804 (2019).
5. Toth, J.J., Pierson, B.D., Painter, C.L., Greenwood, L.R., Burns, K.A., Love, E.F., Soderquist, C.Z., Lavender, C.A., Wall, D.E., Wittman, R.S. Production of Molybdenum-99 Using Neutron Capture Methods; Technical report; PNNL-19895, RPT-59331-01 Rev 0; Pacific Northwest National Laboratory (PNNL): Richmond, WA, USA, (2011).
6. Svoboda, K. Survey of solvent extraction 99mTc-generator technologies. *Radiochim. Acta*. 41(2-3), 83–90 (1987).
7. Endpoint News. Novartis' radiopharma drug wins approval in tough-to-treat prostate cancer patients (accessed 12 Sep. 2022) https://endpts.com/novartis-radiopharma-drug-wins-approval-in-tough-to-treat-prostate-cancer-patients/
8. Fierce Pharma. Novartis accused of gaming Europe's orphan program to price Lutathera sky-high (accessed 12 Sep. 2022) https://www.fiercepharma.com/pharma/novartis-accused-gaming-ema-orphan-program-to-price-lutathera-price-sky-high
9. Czernin, J. Personalized nuclear medicine: the cost of it all. Presentation for Nuclear Medicine Europe (NMEU) Symposium, (2022). https://brolbrolbrol.com/aipessymposium_wp/wp-content/uploads/2021/11/03_Czernin_NMEU_Symposium_2021.pdf
10. Bauer-Nilsen, K., Hill, C., Trifiletti, D. M., Libby, B., Lash, D. H., Lain, M. Showalter, T. N., *et. al.* Evaluation of delivery costs for external beam radiation therapy and brachytherapy for locally advanced cervical cancer using time-driven activity-based costing. *Int. J. Rad. Onc. Bio. Phys.* **100(1)**, 88–94 (2018).

11. Dutta, S. W., Bauer-Nilsen, K., Sanders, J. C., Trifiletti, D. M., Libby, B., Lash, D. H., Showalter, T. N., *et. al.* Time-driven activity-based cost comparison of prostate cancer brachytherapy and intensity-modulated radiation therapy. *Brachytherapy*. **17(3)**, 556–563 (2018).
12. Mausolf, E. J., Johnstone, E. V., Mayordomo, N., Williams, D. L., Guan, E. Y. Z., Gary, C. K. Fusion-Based Neutron Generator Production of Tc-99m and Tc-101: A Prospective Avenue to Technetium Theranostics. *Pharmaceuticals*. **14**, 875 (2021). https://doi.org/10.3390/ph14090875
